# Subcellular Localization Screening of *Colletotrichum higginsianum* Effector Candidates Identifies Fungal Proteins Targeted to Plant Peroxisomes, Golgi Bodies, and Microtubules

**DOI:** 10.3389/fpls.2018.00562

**Published:** 2018-05-02

**Authors:** Guillaume P. Robin, Jochen Kleemann, Ulla Neumann, Lisa Cabre, Jean-Félix Dallery, Nicolas Lapalu, Richard J. O’Connell

**Affiliations:** ^1^UMR BIOGER, Institut National de la Recherche Agronomique, AgroParisTech, Université Paris-Saclay, Versailles, France; ^2^Department of Plant Microbe Interactions, Max Planck Institute for Plant Breeding Research, Cologne, Germany; ^3^Central Microscopy, Max Planck Institute for Plant Breeding Research, Cologne, Germany

**Keywords:** fungus, effectoromics, *Colletotrichum*, localization, peroxisome, nucleus, microtubules, Golgi

## Abstract

The genome of the hemibiotrophic anthracnose fungus, *Colletotrichum higginsianum*, encodes a large inventory of putative secreted effector proteins that are sequentially expressed at different stages of plant infection, namely appressorium-mediated penetration, biotrophy and necrotrophy. However, the destinations to which these proteins are addressed inside plant cells are unknown. In the present study, we selected 61 putative effector genes that are highly induced in appressoria and/or biotrophic hyphae. We then used *Agrobacterium*-mediated transformation to transiently express them as *N*-terminal fusions with fluorescent proteins in cells of *Nicotiana benthamiana* for imaging by confocal microscopy. Plant compartments labeled by the fusion proteins in *N. benthamiana* were validated by co-localization with specific organelle markers, by transient expression of the proteins in the true host plant, *Arabidopsis thaliana*, and by transmission electron microscopy-immunogold labeling. Among those proteins for which specific subcellular localizations could be verified, nine were imported into plant nuclei, three were imported into the matrix of peroxisomes, three decorated cortical microtubule arrays and one labeled Golgi stacks. Two peroxisome-targeted proteins harbored canonical *C*-terminal tripeptide signals for peroxisome import *via* the PTS1 (peroxisomal targeting signal 1) pathway, and we showed that these signals are essential for their peroxisome localization. Our findings provide valuable information about which host processes are potentially manipulated by this pathogen, and also reveal plant peroxisomes, microtubules, and Golgi as novel targets for fungal effectors.

## Introduction

Filamentous plant pathogens such as oomycetes and fungi establish disease by secreting an array of effector proteins that manipulate plant processes and mitigate plant immune responses to create a favorable environment for pathogen growth (Dodds and Rathjen, 2010). Following their secretion from infection structures such as appressoria, hyphae, and haustoria, effector proteins exert their biological activity either outside plant cells (in the plant apoplast and/or plant–pathogen interface) or inside the plant cytoplasm after translocation across the plant plasma membrane (Giraldo and Valent, 2013; Lo Presti et al., 2015).

A major challenge for the functional analysis of effectors from filamentous plant pathogens is the large number of candidate proteins (several hundred) encoded in each oomycete or fungal genome (Dong et al., 2015). For translocated (cytoplasmic) effectors, knowledge of the plant subcellular compartments to which they are targeted can give valuable insights into the plant processes and proteins that they potentially interact with, and facilitates the prioritization of candidates for functional analysis (Petre et al., 2015). Medium-throughput screens have been developed to systematically localize effectors *in planta*, based on the transient expression of effectors as translational fusions with fluorescent proteins (FPs) in *Nicotiana benthamiana* leaf cells followed by confocal microscopy (Boevink et al., 2011). To date, cell biology screens of this type have been implemented with effector libraries from the oomycetes *Phytophthora infestans* and *Hyaloperonospora arabidopsidis* and two species of rust fungi, *Melampsora larici-populina* and *Puccinia striiformis* f. sp. *tritici* (Schornack et al., 2010; Caillaud et al., 2012; Petre et al., 2015, 2016). The plant compartments targeted by effectors from these four pathogens included nuclei, chloroplasts, ER, tonoplast, and plasma membranes.

*Colletotrichum* is a large ascomycete genus comprising 190 species, many of which cause devastating diseases on numerous agricultural and horticultural crops world-wide (Crouch et al., 2014; Jayawardena et al., 2016). The crucifer anthracnose pathogen, *C. higginsianum*, is economically important on cultivated brassicas, but also attacks the model plant *Arabidopsis thaliana*, providing a pathosystem in which both partners can be genetically manipulated (Narusaka et al., 2004; O’Connell et al., 2004). Similar to other members of the genus, *C. higginsianum* employs a ‘hemibiotrophic’ infection strategy (Crouch et al., 2014): after melanized appressoria breach the host cuticle and cell wall, the fungus initially grows inside living host cells without causing visible disease symptoms. During this initial biotrophic phase, the bulbous intracellular hyphae are tightly enveloped by a modified region of the plant plasma membrane (O’Connell et al., 2004; Shimada et al., 2006). Later, the pathogen switches to a destructive necrotrophic lifestyle associated with rapid host cell death and the maceration of host tissues.

Analysis of the genomes and *in planta* transcriptomes of several *Colletotrichum* species has uncovered large inventories of genes (150–350 per genome) encoding putative secreted effector proteins of unknown function (Kleemann et al., 2012; O’Connell et al., 2012; Gan et al., 2013; Bhadauria et al., 2015; Baroncelli et al., 2016). During plant infection, distinct subsets of effector genes are expressed in successive waves associated with appressorial penetration, biotrophic intracellular growth and the switch to necrotrophy (Kleemann et al., 2012; Gan et al., 2013). Biological functions have been ascribed to relatively few *Colletotrichum* effectors. Two chitin-binding LysM domain effectors from *C. higginsianum*, ChELP1 and ChELP2, suppress chitin-triggered immune responses and are also required for appressorial penetration (Takahara et al., 2016). Effectors that elicit plant cell death when expressed in *N. benthamiana* were identified from several *Colletotrichum* species, for example *C. orbiculare* NIS1, the Nep1-like protein NLP1 from *C. higginsianum*, and the nudix hydrolase domain-containing CtNUDIX from *C. lentis* (Kleemann et al., 2012; Yoshino et al., 2012; Bhadauria et al., 2013). Gene deletion experiments showed that CgDN3 from *C. gloeosporioides* functions to suppress host cell death during the infection of *Stylosanthes guianensis* (Stephenson et al., 2000). Similarly, the homologous effectors from *C. higginsianum* (ChEC3 and ChEC3a) and *C. orbiculare* (CoDN3) suppressed plant cell death elicited by NLP1 and NIS1, respectively, when co-expressed in *N. benthamiana* (Kleemann et al., 2012; Yoshino et al., 2012). Proteins of the DN3 family appear to be cytoplasmic effectors because they retain their cell death suppression activity when expressed in plant cells without a signal peptide (Kleemann et al., 2012; Yoshino et al., 2012). The effectors CoMC69 from *C. orbiculare* and CgEP1 from *C. graminicola* were found to be essential for fungal virulence but their biological functions remain unknown (Saitoh et al., 2012; Vargas et al., 2016).

In *C. higginsianum* and *C. orbiculare*, effectors have been localized during infection by expressing them in the fungus as fusions with FPs (Kleemann et al., 2012; Irieda et al., 2014) or using antibodies raised to the native protein for immunocytochemistry (Takahara et al., 2016). These studies revealed that early-expressed effectors are concentrated inside appressorial pores before host penetration; whereas those expressed after penetration accumulate at the plant–fungal interface around biotrophic hyphae, and in some cases become concentrated in small punctae termed interfacial bodies (Kleemann et al., 2012), or in ring-shaped accumulations around the necks of biotrophic hyphae (Irieda et al., 2014). By analogy to other pathogens, it is assumed that some *Colletotrichum* effectors act inside the plant cytoplasm. However, after FP-tagging six *C. higginsianum* effectors and three *C. orbiculare* effectors, none were detectable inside the host cytoplasm (Kleemann et al., 2012; Irieda et al., 2014). To date, the only direct evidence for translocation of a *Colletotrichum* effector comes from *C. graminicola*, where the FP-tagged effector CgEP1 was detected in host nuclei after secretion by the fungus (Vargas et al., 2016). CgEP1 carries a predicted nuclear localization signal (NLS) that is expected to concentrate the fusion protein in host nuclei, thereby enhancing fluorescence detection sensitivity (Giraldo and Valent, 2013). The failure to detect other FP-tagged *Colletotrichum* effectors inside host cells could result from the amount of translocated fusion protein being below the detection limit of confocal microscopy (Lo Presti et al., 2015). Alternatively, the relatively large FP tag may interfere with translocation of the fusion protein across the host plasma membrane, although the mechanism by which fungal effectors traverse this membrane remains poorly understood (Petre and Kamoun, 2014; Lo Presti et al., 2015).

In the present study, we selected sixty-one *C. higginsianum* genes encoding putative effectors that are highly induced in penetrating appressoria and/or in biotrophic hyphae and cloned them into a plant expression vector providing an *N*-terminal GFP tag. We used *Agrobacterium*-mediated transformation to transiently express the tagged proteins directly inside plant cells and then localized them by confocal microscopy. This revealed that nine *C. higginsianum* effector candidates (ChECs) were specifically imported into plant nuclei, one labeled plant Golgi bodies, three were imported into the matrix of plant peroxisomes, while three others decorated plant cortical microtubules. To our knowledge, plant Golgi, peroxisomes and microtubules were not previously reported to be targets for the effectors of any other filamentous plant pathogens. Moreover, two of the peroxisome-targeted proteins contain canonical *C*-terminal tripeptide signals for peroxisome import *via* the PTS1 pathway, and by deleting these signals we validated that they are essential for peroxisome localization. Our study shows that multiple *C. higginsianum* effectors converge on plant peroxisomes and microtubules, suggesting that fungal manipulation of host functions associated with these structures may be critical for successful pathogenesis.

## Materials and Methods

### Synthesis and Cloning of Putative *C. higginsianum* Effector Genes

Nucleotide sequences for gene synthesis were designed from the predicted cDNA sequences of ChECs that were previously identified from ESTs and the fungal genome sequence (Kleemann et al., 2012; O’Connell et al., 2012). Selected ChEC genes were synthesized using GeneART^®^ gene synthesis technology without their predicted fungal signal peptides and cloned into the pDONR221 GATEWAY entry vector (Invitrogen, Darmstadt, Germany). The sequences were then transferred using the LR cloning reaction into binary destination vectors derived from pSITE (Martin et al., 2009) for transient expression of the proteins in plants as *N*-terminal fusions with EGFP (pSITE-2CA) or mRFP (pSITEII-6C1). After the LR reaction, the constructs were cloned into *Escherichia coli* TOP10 cells and then *Agrobacterium tumefaciens* (C58C1, pGV2260, or pMP90). *E. coli* cells were cultivated at 37°C in lysogenic broth (LB) medium (Bertani, 1951). *A. tumefaciens* strains were cultivated on LB at 28°C, with appropriate antibiotic selection (spectinomycin 100 μg ml^-1^, streptomycin 50 μg ml^-1^, rifampicin 50 μg ml^-1^).

### Expression Profiling of Putative Effector Genes

The expression patterns of the selected ChEC genes were profiled using RNA-Seq data corresponding to four developmental stages of *C. higginsianum*, namely *in vitro* appressoria (22 h post inoculation, hpi), *in planta* appressoria (22 hpi), biotrophic phase (40 hpi), and necrotrophic phase (60 hpi). Preparation of the RNA samples was described previously (O’Connell et al., 2012) and the raw data sets are available under GEO accession GSE33683. Here, the filtered reads were mapped onto the reannotated genome assembly of *C. higginsianum* (Dallery et al., 2017) using TopHat2 (Kim et al., 2013) (version 2.0.14, *I* = 5000, *a* = 10, *g* = 5). Previously described ChECs (Kleemann et al., 2012) that lack a gene model in the new annotation were manually incorporated into the annotation file used for mapping. HTseq (Anders et al., 2015) (version 0.5.3p9) was used to count the mapped reads, and ‘Relative Expression Index’ and gene expression level were calculated as described previously (Hacquard et al., 2016). Heatmaps were produced using Genesis software, version 1.7.6 (Sturn et al., 2002).

### Transient Expression of Proteins in *N. benthamiana* Leaves

*Nicotiana benthamiana* plants were grown under long-day conditions (16 h light, 25°C, 80% relative humidity; 8 h dark, 22°C, 50% relative humidity). An over-night culture of *A. tumefaciens* strain C58C1 pGV2260 was centrifuged, resuspended in buffer (10 mM MgCl_2_, 10 mM MES-KOH pH 5.7, 150 μM acetosyringone) and incubated for 3 h at room temperature. The bacterial suspension was adjusted to OD_600__nm_ = 0.4 and pressure-infiltrated into the abaxial surface of *N. benthamiana* leaves of 4-week-old plants using a needleless syringe (1 ml). For co-expression experiments, Agrobacteria harboring each construct were mixed and adjusted to OD_600__nm_ = 0.4 in the same suspension and infiltrated. To ensure high levels of transient expression of both constructs in these experiments, we also co-expressed the P19 gene, a suppressor of post-transcriptional gene silencing from the *Tomato bushy stunt virus* (Oh et al., 2009). Agrobacteria harboring P19 were infiltrated at a final OD_600__nm_ of 0.1. At 24–48 h after agro-infiltration, pieces of leaf tissue (∼5 mm × 5 mm) were excised for examination by confocal microscopy (described below).

### Transient Expression of Proteins in *A. thaliana* Seedlings

The protocol was adapted from that of Marion et al. (2008). Briefly, *A. thaliana* seeds were surface-sterilized by washing in ethanol (70% v/v) for 1 min and sodium hypochlorite (commercial bleach, 3% v/v) for 15 min and then rinsed five times in sterile water. Seeds were then placed into the wells of a sterile 6-well plate, each well containing 4 ml of half-strength Murashige and Skoog medium covered with a sterile nylon mesh disk (200 μm mesh size). Plates were maintained at 4°C overnight before being transferred to a controlled environment chamber (12 h photoperiod, 230 μmole m^-2^ s^-1^ photon flux density, 23°C). Transformation was performed on 4-day-old seedlings. An over-night culture of *A. tumefaciens* strain C58C1 pMP90 was centrifuged and resuspended in buffer (10 mM MgCl_2_, 10 mM MES-KOH pH 5.7, 150 μM acetosyringone) and incubated for 3 h at room temperature. Seedlings were completely immersed in the bacterial suspension and then vacuum-infiltrated twice for 1 min. The bacterial suspension was then removed by pipetting and the plants transferred to a growth chamber for a further 3 days. Cotyledons were excised prior to mounting in water under a coverslip for confocal microscopy.

### Confocal Microscopy

To determine the subcellular localization of the GFP-tagged ChECs in *N. benthamiana* leaf pieces or *A. thaliana* seedlings, samples were first mounted under a coverslip in water inside glass-bottomed culture dishes (40 mm diameter, 0.17 mm glass thickness, WillCo-Dish^®^) and then observed using either Leica SPE or Leica SP5 inverted confocal microscopes equipped with a ×63 (1.2 NA) water immersion objective. To image GFP fluorescence, the 488 nm laser line was used for excitation and emission was collected between 490 and 525 nm. For imaging mRFP fluorescence, excitation was at 532 nm and emission was observed between 580 and 650 nm. To minimize spectral bleed-through between fluorescence channels during co-localization experiments, images were acquired by sequential scanning with alternation between frames (Leica SPE microscope) or between lines (Leica SP5 microscope). Plant nuclei were stained using DAPI (4’,6-diamidino-2-phenylindole, 10 μg/ml in water) pressure-infiltrated into the abaxial side of the leaf using a 1 ml syringe 30 min before observation. DAPI fluorescence was excited at 405 nm and observed between 440 and 475 nm. Measurement of nuclear areas was performed on maximum projections of confocal image stacks using the Fiji particle analysis tool after manual thresholding (Schindelin et al., 2012). Incomplete nuclei located at image boundaries were manually excluded from these analyses.

### Transmission Electron Microscopy

Pieces (2 mm × 3 mm) of *N. benthamiana* leaves (2 days after agro-infiltration) were fixed for 2 h in a mixture of 4% (w/v) para-formaldehyde and 0.5% (v/v) glutaraldehyde in 0.05 M sodium cacodylate buffer (pH 6.9). After dehydration in a graded water–ethanol series, the samples were embedded in LR White acrylic resin using the progressive lowering of temperature method (Satiat-Jeunemaitre and Hawes, 1992). Adjacent ultrathin sections were mounted on separate specimen grids for labeling with different antibodies. Immunogold labeling was as described by Kleemann et al. (2012) except that GFP was detected using a rabbit polyclonal anti-GFP antibody (ab6556, Abcam, diluted 1:50) and DsRed was detected using a rabbit polyclonal anti-RFP antibody (R10367, Molecular Probes, diluted 1:250). Goat anti-rabbit antibodies conjugated with 10 nm colloidal gold particles (British Biocell International, Cardiff, United Kingdom) were used as secondary antibodies. Sections were stained with 2% uranyl acetate for 10 min followed by lead citrate for 15 min and examined with an Hitachi H-7650 TEM operating at 100 kV fitted with an AMT XR41 M digital camera.

### Total Protein Isolation and Immunoblot Assays

Agro-infiltrated *N. benthamiana* leaves (2 days after infiltration) were first checked for expression of GFP-ChEC fusion proteins by epi-fluorescence microscopy and then snap-frozen in liquid nitrogen. The frozen leaves were reduced to powder using a cold mortar and pestle. Protein extracts were prepared as described by Petre et al. (2015) and 15 μl samples were run on 12% SDS-PAGE gels. Protein concentrations were estimated by Ponceau staining. Proteins separated by SDS-PAGE were transferred to polyvinylidene difluoride membranes (Bio-Rad #1620177) using a *Trans*-blot Semi Dry transfer cell (Bio-Rad) in transfer buffer (Tris 25 mM, glycine 192 mM, ethanol 20% v/v, SDS 0.1% w/v, pH 8.3). After blocking in 3% w/v bovine serum albumin (BSA) in TBST (Tris-buffered saline containing 0.1% v/v Tween 20), the membranes were probed with mouse anti-GFP monoclonal antibodies (Santa Cruz F56-6A1) diluted 1:5,000 in 3% BSA for 1 h, followed by goat anti-mouse polyclonal antibodies conjugated with horse radish peroxidase (HRP, Dako P0447) diluted 1:20,000 in 3% BSA for 1 h. Blots were developed using Immobilon Western HRP substrate (Millipore) for chemiluminescence detection.

## Results

### Identification, Curation, and Cloning of *C. higginsianum* Effector Candidates

**FIGURE 1:**
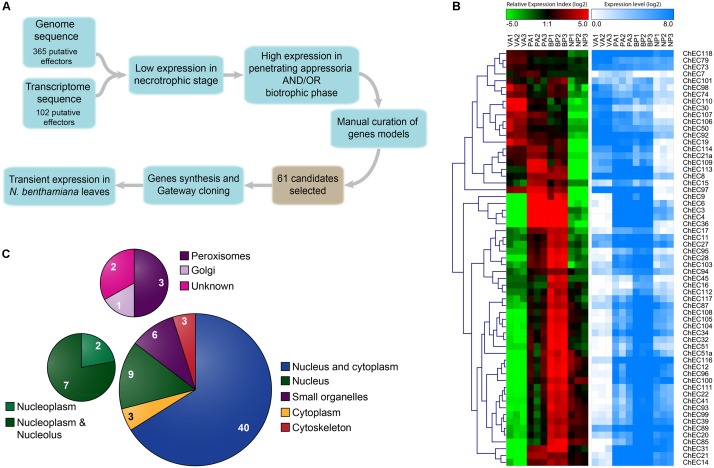
Identification and selection of *C. higginsianum* effector candidates (ChECs). **(A)** Workflow and criteria used for the selection of 61 ChECs from the effector repertoires previously predicted from the fungal genome and *in planta* transcriptome (Kleemann et al., 2012; O’Connell et al., 2012). The cDNA sequences of the selected ChECs were synthesized and cloned into a Gateway entry vector before sub-cloning into a destination vector providing an *N*-terminal GFP tag for transient expression in *N. benthamiana*. **(B)** Heatmaps showing the gene expression profiles of 61 ChEC genes across the four developmental stages selected for RNA sequencing (VA, *in vitro* appressoria; PA, *in planta* appressoria; BP, biotrophic phase; NP, necrotrophic phase). Over-represented (dark red to bright red) or under-represented transcripts (dark green to bright green) are shown as log_2_ Relative Expression Index values. In addition, log_2_ gene expression levels are displayed as a gradient from white (no expression) to blue (high expression). **(C)** Pie chart summarizing the distribution of GFP-tagged ChECs between different compartments of *N. benthamiana* cells, as determined by confocal microscopy. While the majority (43) showed either a cytoplasmic or nucleo-cytoplasmic localization, others were preferentially or specifically localized in the plant nucleus (9), plant organelles (6), or on elements of the plant cytoskeleton (3).

We previously identified genes encoding potential effector proteins from *C. higginsianum* using two independent approaches. Based on the fungal genome annotation, we identified 365 Candidate Secreted Effector Proteins (CSEPs) defined as extracellular proteins (WoLF PSORT prediction) without homology to proteins from organisms outside the genus *Colletotrichum* (O’Connell et al., 2012). Based on mining EST data from deep-sequencing *in planta* cDNA libraries, we found 102 ChECs based on the presence of an *N*-terminal signal peptide (SignalP prediction) and their lack of homology to known proteins, or similarity to effectors from other fungi (Kleemann et al., 2012) (**Figure 1A**). In the present study, data from both approaches were combined to obtain a non-redundant list of effectors, hereafter referred to as ChECs. We selected candidates that were preferentially expressed *in planta* by appressoria and/or biotrophic hyphae by filtering available RNA-Seq data (O’Connell et al., 2012) to include only genes with <15% of total reads derived from a necrotrophic phase library and >50% reads derived from penetrating appressoria and/or biotrophic phase libraries. Genes with low expression levels were discarded if the total number of mapped reads across all three *in planta* infection stages was <50. Finally, all gene models were manually curated by comparison to the transcriptome data to correct errors in start/stop sites, intron structure and sequence indels. Following this procedure, a total of 61 ChECs were selected for further study (**Figure 1A** and Supplementary Table 1).

To search for the presence of homologous proteins in other fungi, we blasted the protein sequences of the 61 ChECs against the UniProt database (Swiss-Prot + trEMBL, version 02/03/2018) using blastp. Sequences with an *e*-value < 1 × 10^-3^, identity >25%, and coverage >75% were considered as potential homologs. On this basis, we found that 20 ChECs were “species-specific” with no homolog in other *Colletotrichum* species, 30 were “genus-specific” with homolog(s) limited to the genus *Colletotrichum* and 11 were “non-specific” with homolog(s) in other fungal genera (Supplementary Table 2). For the latter category, homologs were found most often in *Fusarium oxysporum*, *Ceratocystis fimbriata* and in species of *Diaporthe* and *Rhynchosporium.*

Expression profiles of the selected ChEC genes across four fungal developmental stages are presented in **Figure 1B**, based on mapping previous RNA-Seq data (O’Connell et al., 2012) to a revised assembly and annotation of the *C. higginsianum* genome (Dallery et al., 2017). Approximately two-thirds of the ChEC genes appear to be plant-induced, because they showed minimum expression in appressoria formed *in vitro*. Consistent with our previous qRT-PCR analysis of 17 ChECs (Kleemann et al., 2012), the 61 genes selected for study here also showed highly stage-specific expression. Thus, while 21 genes were preferentially expressed in appressoria (‘wave 1’), only eight were induced in both appressoria and the biotrophic phase (‘wave 2’), and the largest group (32 genes) were preferentially expressed during the biotrophic phase (‘wave 3’).

### Mapping the Destinations of Fungal Effectors Expressed Inside Plant Cells

The predicted cDNA sequences of the selected ChECs were synthesized and cloned into a Gateway entry vector. In each case, the fungal signal peptide was omitted from the 5′ end, while adding an artificial start codon and retaining the original stop codon. The cloned ChEC sequences were shuttled into a binary destination vector suitable for transient over-expression of the proteins in *N. benthamiana* leaves via *Agrobacterium* infiltration. The vector provided a fusion to GFP at the *N*-terminus of each protein and expression was driven from the 35S promoter. After 2–3 days, the Agro-infiltrated tissues were examined by confocal microscopy to determine the subcellular localization of the fusion proteins.

**FIGURE 2:**
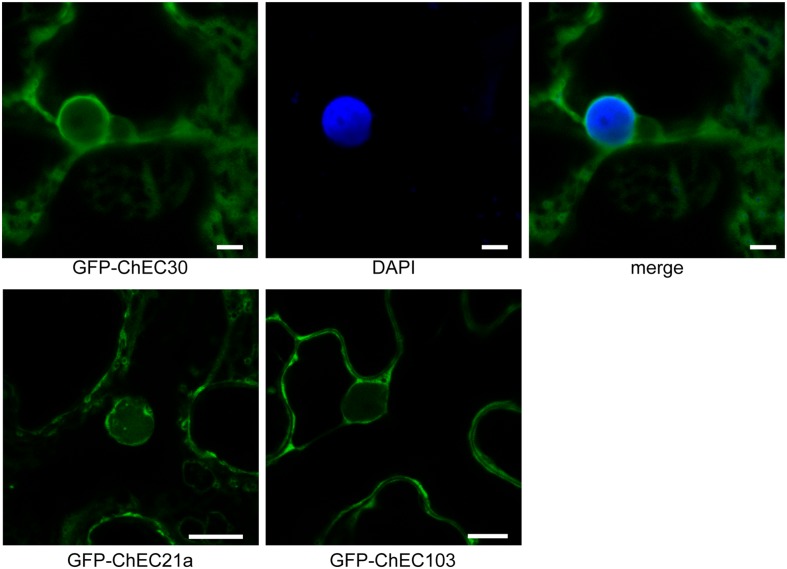
Confocal micrographs showing that GFP-tagged ChECs (ChEC30, ChEC21a, and ChEC103) are excluded from plant nuclei and are confined to the cytosol upon transient expression in *N. benthamiana* cells. In the case of GFP-ChEC30 the plant nucleus is stained blue with DAPI. Bars = 5 μm.

Among the 61 GFP-tagged ChECs screened in this assay, 40 showed no specific localization in that they diffused throughout the plant nucleoplasm and cytoplasm, a pattern that was indistinguishable to that of GFP expressed alone (**Figure 1C** and Supplementary Figure 1). However, the remaining 21 fusion proteins were targeted either specifically or preferentially to particular compartments of the plant cell. Thus, nine GFP-ChECs were targeted to the plant nucleus, of which eight preferentially accumulated in the nucleolus, six labeled small punctate structures that were likely to be plant organelles, while three others decorated filamentous structures that resembled elements of the plant cytoskeleton (**Figure 1C**). A further three ChECs were excluded from the plant nucleus and confined to the plant cytoplasm (**Figure 2**). Their exclusion from the nucleus appears unrelated to their size because the predicted molecular weights (MW) of the mature proteins (ChEC21a = 10.38 kDa, ChEC30 = 16.78 kDa, ChEC103 = 6.99 kDa) were only slightly greater, or less than, the mean ChEC MW (11.45 kDa, Supplementary Table 1). In the case of ChEC30 and ChEC103, this localization was consistent with the detection of a putative nuclear export signal using the NetNES prediction tool (La Cour et al., 2004).

The subcellular localizations determined experimentally by GFP-tagging the ChECs were compared with localizations predicted by the LOCALIZER tool (Sperschneider et al., 2017) running in ‘effector mode’ or by WoLF PSORT (Horton et al., 2007) running in ‘plant mode’ after removing the fungal secretion signal. The results are compiled in Supplementary Table 1. LOCALIZER correctly predicted eight ChECs to be nuclear-targeted but eight others were false positives and one other was not predicted to be nuclear (false negative). LOCALIZER is currently not able to predict Golgi, peroxisomes or cytoskeleton as protein destinations. WoLF PSORT correctly predicted six nuclear-targeted ChECs with seven false positives and one false negative. WoLF PSORT predicted a chloroplast localization for no fewer than 15 of the ChECs (all incorrect based on our experimental results), while Golgi and cytoskeleton were not predicted as destinations for any of the proteins.

The integrity of the GFP fusion proteins was verified by Western blotting using anti-GFP antibodies. For all the ChECs showing a specific subcellular localization (21), we were able to confirm the presence of the full-length fusion protein without detectable free GFP, suggesting that cleavage of the FP from the effector had not occurred. We likewise verified the integrity of six GFP-ChECs that showed a nucleo-cytoplasmic localization, again confirming that their distribution was not due to cleavage and subsequent diffusion of free GFP (Supplementary Figure 2).

### Numerous *C. higginsianum* Effector Candidates Are Imported Into Plant Nuclei

**FIGURE 3:**
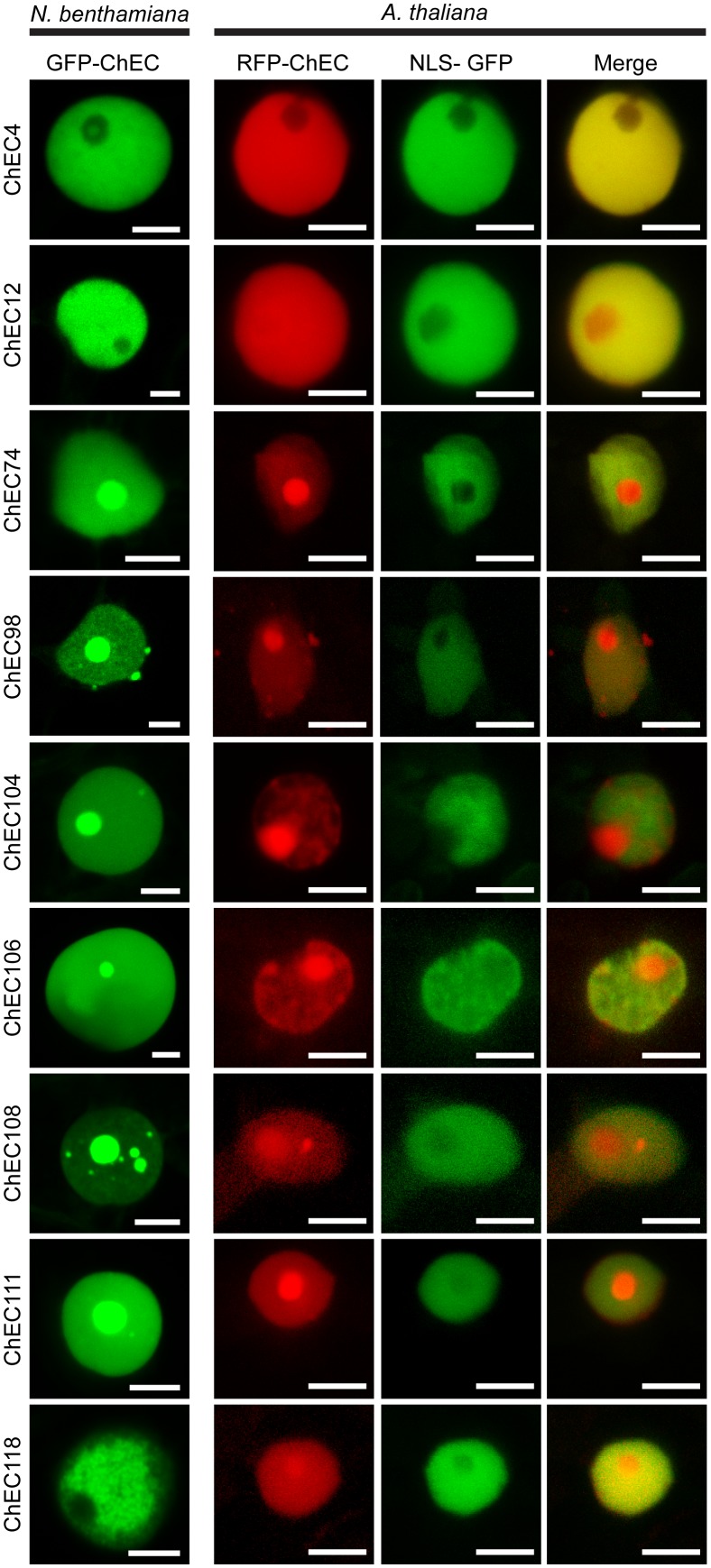
Confocal microscope z-stack projections showing the localization patterns of nine ChECs in plant nuclei. **(Left)** GFP-tagged ChECs were transiently expressed in *N. benthamiana* leaf cells. Bars = 10 μm. **(Right)** RFP-tagged ChECs were transiently expressed in transgenic *A. thaliana* seedlings stably expressing an NLS-GFP-GUS fusion protein to label plant nuclei. Bars = 10 μm.

Nine ChECs were localized in the nuclei of *N. benthamiana* cells. To confirm this localization in the true host of *C. higginsianum*, we transiently expressed the proteins as *N*-terminal fusions with mRFP in transgenic *A. thaliana* seedlings stably expressing GFP fused to β-glucuronidase (GUS) and an NLS, which accumulates specifically in nuclei (Chytilova et al., 1999). All nine effector candidates that were imported into *N. benthamiana* nuclei also accumulated in *A. thaliana* nuclei (**Figure 3**). To further support these *in situ* localization data, we used four different computational algorithms to search for the presence of putative nuclear localization signals (NLS) within the predicted sequences (minus signal peptide) of these proteins, namely NLSpredict (Yachdav et al., 2014), cNLS Mapper (Kosugi et al., 2009), NLStradamus (Nguyen Ba et al., 2009), and WoLF PSORT (Horton et al., 2007). All nine of the nuclear-targeted ChECs possessed an NLS that was predicted by two or more different algorithms (Supplementary Table 3). In contrast, among the remaining 52 ChECs that did not accumulate in plant nuclei, only four were predicted to contain an NLS by two or more algorithms (ChEC9, ChEC51, ChEC96, and ChEC113).

Interestingly, the precise distribution of these nine ChECs in plant nuclei was not identical, and we were able to distinguish several different patterns. ChEC4 labeled the nucleoplasm but was excluded from the nucleolus in both *N. benthamiana* and *A. thaliana* (**Figure 3**). ChEC118 did not label the *N. benthamiana* nucleoplasm uniformly, instead showing a granular pattern reminiscent of chromatin structure (**Figure 3**). When GFP-ChEC118-labeled nuclei were in addition stained with DAPI, we observed that some regions of the nucleoplasm that were enriched with GFP-ChEC118 were not stained by DAPI (Supplementary Figure 3). This partitioning of the plant DNA contrasts to normal *N. benthamiana* nuclei at interphase, where DAPI labels the nucleoplasm uniformly (Supplementary Figure 4). However, this partitioning of the nucleoplasm was not visible in nuclei of *A. thaliana* cells expressing RFP-ChEC118 (**Figure 3**).

Six other nuclear-targeted proteins, namely ChEC74, ChEC98, ChEC104, ChEC106, ChEC108, and ChEC111, were concentrated inside the nucleolus and other sub-nuclear compartments in both *N. benthamiana* and *A. thaliana* (**Figure 3**). Punctate accumulations of ChEC98 of varying size were also present on the surface of nuclei in both plant species. These structures do not resemble known compartments of the plant cell and may represent artefactual protein aggregates caused by over-expression of the fusion protein. ChEC74 and ChEC104 had similar localization patterns in that they preferentially labeled nucleoli more than the nucleoplasm, and both were predicted to harbor a nucleolar targeting signal using the NOD predictor (Scott et al., 2010, 2011). In addition to being concentrated in the nucleolus, ChEC108 also labeled smaller sub-nuclear structures that resembled Cajal bodies in size (0.2–2 μm) and number (1–6 per nucleus) in both *N. benthamiana* and *A. thaliana* (**Figure 3**). In order to identify these compartments, we co-expressed GFP-ChEC108 or RFP-ChEC108 together with protein markers labeling the nucleolus and Cajal bodies, namely Fibrillarin 2 (RFP-FIB2) and U2 Small nuclear ribonucleoprotein B (GFP-U2B; Shaw et al., 2014). A sub-set of small ChEC108-positive structures co-localized with FIB2 or U2B, and are therefore likely to be Cajal bodies (Supplementary Figure 5). However, other sub-nuclear compartments were only labeled by ChEC108 and therefore remain unidentified.

Remarkably, and in contrast to all the other nuclear-targeted ChECs, transient over-expression of GFP-ChEC106 in *N. benthamiana* resulted in an increase in the size of the nucleus in transformed cells, involving an inflation of the nucleoplasm but not the nucleolus (**Figure 3** and Supplementary Figure 4). Thus, the size of nuclei in cells expressing GFP-ChEC106 (mean area = 175.1 μm^2^, *n* = 106) was 2.2-fold larger than in cells expressing GFP-ChEC104 (mean area = 80.0 μm^2^, *n* = 49) and 2.9-fold larger than in untransformed cells, in which nuclei were stained with DAPI (mean area = 59.6 μm^2^, *n* = 81) (Supplementary Figure 4D). The observed differences were highly significant in all pair-wise comparisons (Mann–Whitney non-parametric test, *p* < 0.01). The nuclei of transformed cells did not appear enlarged in *A. thaliana* seedlings expressing RFP-ChEC106 (**Figure 3**), although nuclear sizes were not quantified.

### Identification of Putative Plant Organelles Labeled by ChECs

Six of the GFP-ChECs became concentrated in small punctate structures moving rapidly in the plant cytoplasm, which could represent plant organelles. None of these were chloroplasts, which could be easily distinguished by their larger size and chlorophyll autofluorescence. To identify the labeled compartments, we performed co-localization experiments by transiently co-expressing the GFP-ChECs in *N. benthamiana* cells together with organelle-specific markers tagged with red FPs. Peroxisomes were labeled with PTS2-DsRed (peroxisome targeting signal 2 fused to DsRed; Fujimoto et al., 2009). Mitochondria were labeled with Mt-RFP (mitochondrial targeting signal of the *Arabidopsis* ATPase δ-subunit fused to mRFP (Arimura and Tsutsumi, 2002). The *trans*-Golgi cisternal membranes of Golgi stacks were labeled with ST-mRFP (signal anchor of rat sialyltransferase; Sparkes et al., 2006; Uemura et al., 2012). Early and late endosomes, respectively, were labeled using the Rab-like GTPases ARA6 and ARA7 fused to RFP (Ueda et al., 2001). Finally, the *trans*-Golgi network/early endosome compartment was labeled with the syntaxin Syp61 fused to RFP (Choi et al., 2013). Among the GFP-ChECs labeling putative plant organelles, ChEC36 and ChEC39 did not co-localize with any of the six organelle markers and labeled punctate structures of two different sizes (mean diameter 0.98 μm and 2.15 μm for ChEC36 and ChEC39, respectively, *n* = 36) (Supplementary Figure 6).

**FIGURE 4:**
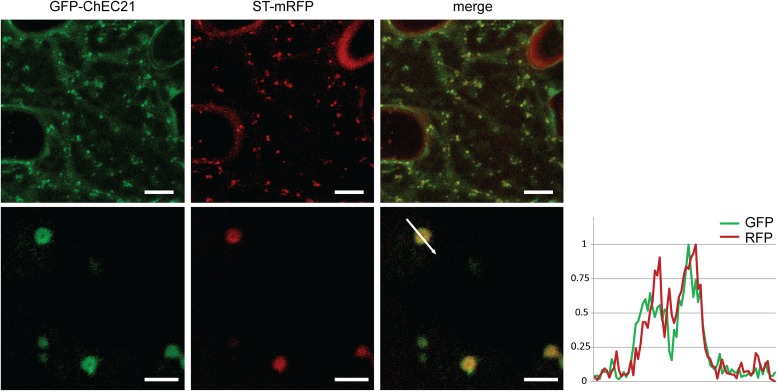
GFP-tagged ChEC21 is addressed to plant Golgi stacks upon transient expression in *N. benthamiana* cells. GFP-ChEC21 co-localizes with ST-mRFP, a marker for the *trans* face of plant Golgi stacks. The right panel shows a fluorescence intensity plot across one ring-shaped Golgi stack (white arrow) confirming co-localization of the two markers, with bright peripheral labeling around a darker core. Upper panels: bars = 10 μm; lower panels: bars = 2.5 μm.

GFP-ChEC21 perfectly co-localized together with ST-mRFP, consistent with this protein being associated with plant Golgi stacks (**Figure 4**). At higher magnification, the organelles labeled by GFP-ChEC21 and ST-mRFP could be resolved as distinct ring-shaped structures with a bright periphery and a dark core (**Figure 4**), as reported previously for Golgi stacks labeled by ST-GFP and cellulose synthase CESA3-GFP (Boevink et al., 1998; Crowell et al., 2009). This characteristic doughnut-like labeling pattern suggests that ChEC21 labels the rim of plant Golgi stacks.

**FIGURE 5:**
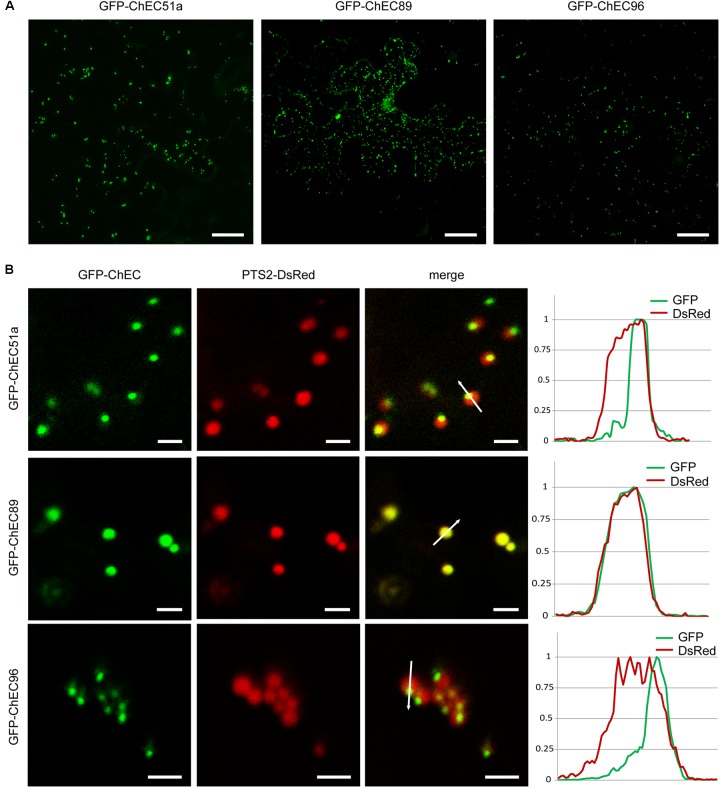
GFP-tagged ChECs targeted to plant peroxisomes upon transient expression in *N. benthamiana* cells. **(A)** GFP-ChEC51a, GFP-ChEC89, and GFP-ChEC96 label small, highly mobile plant organelles. Bars = 25 μm. **(B)** All three fusion proteins co-localize with a PTS2-DsRed marker specific for plant peroxisomes. The right panels are fluorescence intensity plots across representative peroxisomes (white arrows) showing that while ChEC89 is distributed uniformly through the peroxisome matrix, ChEC51a and ChEC96 are concentrated in small punctate inclusions apparently located inside the matrix. Bars = 2.5 μm.

**FIGURE 6:**
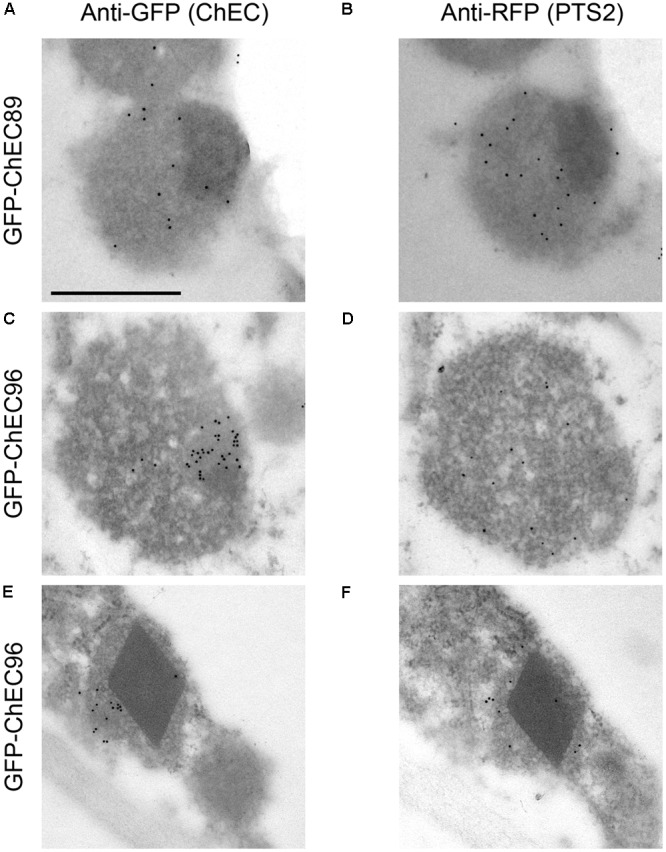
Transmission electron micrographs showing immunogold labeling of *N. benthamiana* peroxisomes in cells co-expressing PTS2-DsRed together with either GFP-CHEC89 **(A,B)** or GFP-ChEC96 **(C–F)**. In each case, adjacent ultrathin sections through the same peroxisome were probed with either anti-GFP antibodies to label the GFP-tagged ChECs or anti-RFP antibodies to label the peroxisome matrix. All micrographs are presented at the same magnification, bar = 500 nm.

Three other GFP-tagged proteins, namely ChEC51a, ChEC89, and ChEC96, labeled small highly mobile organelles of similar size (**Figure 5A**). All three fusion proteins co-localized with PTS2-DsRed, suggesting that the punctate structures labeled by these ChECs are plant peroxisomes (**Figure 5B**). While ChEC89 filled the entire peroxisome matrix, ChEC51a and ChEC96 were concentrated into a smaller punctate structure that appeared to be located inside the peroxisome (**Figure 5B**). To examine the localization of GFP-ChEC89 and GFP-ChEC96 at higher resolution, we used transmission electron microscopy and immunogold labeling with antibodies specific for RFP to detect the PTS2-DsRed peroxisome marker, and antibodies specific to GFP to label the ChECs. In adjacent ultrathin sections through the same peroxisome, both GFP-ChEC89 and PTS2-DsRed were detected in the peroxisome matrix, with little or no labeling detectable on a large electron-opaque inclusion within the matrix, which probably corresponds to the catalase crystal (**Figures 6A,B**). In contrast, GFP-ChEC96 appeared less uniformly distributed through the peroxisome matrix than either GFP-ChEC89 or PTS2-DsRed, and in some sections the GFP-ChEC96 labeling could be seen concentrated into a small area of the peroxisome matrix (**Figures 6C,D**). This is unlikely to represent the catalase crystal because in those peroxisomes where the crystal was clearly recognizable, it showed little or no labeling with anti-GFP antibodies (**Figures 6E,F**). These immunolabeling experiments therefore confirmed that both ChEC89 and ChEC96 are imported into the peroxisome matrix and that ChEC96, but not ChEC89, becomes concentrated into small inclusions that are distinct from the catalase crystal. Similar immunolocalization experiments were not preformed with ChEC51a because the transient over-expression of this protein in *N. benthamiana* was found to cause necrosis in a subset of the transformed cells.

**FIGURE 7:**
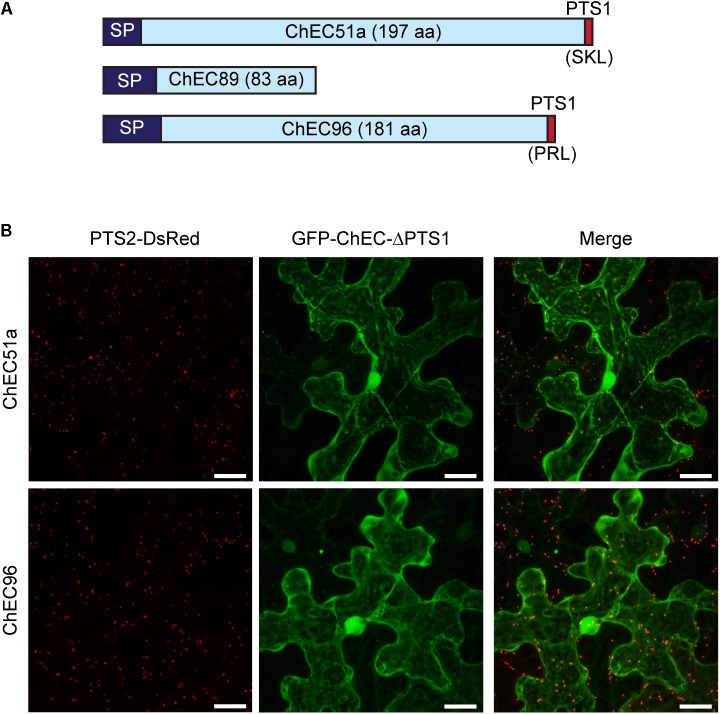
**(A)** Cartoon showing the relative protein size and domain structure of the three peroxisome-targeted effector candidates. SP, signal peptide for secretion; PTS1, peroxisome targeting signal 1. **(B)** Deletion of the *C*-terminal PTS1 signals from GFP-ChEC51a and GFP-ChEC96 abolished the peroxisome localization of both fusion proteins, which instead accumulated in the plant cytosol. Peroxisomes are labeled by co-expression of PTS2-DsRed. Bars = 25 μm.

The import of proteins from the cytosol into the peroxisome matrix is mediated by conserved peroxisome targeting signals, either of type 1 (PTS1) or type 2 (PTS2), which can be predicted using computational tools (Lingner et al., 2011). The plant-specific PredPlantPTS1 algorithm (Reumann et al., 2012) predicted the presence of a PTS1 tripeptide at the *C*-terminus of ChEC51a (SKL, 100% targeting probability) but not in ChEC96 (PRL, 8.7% targeting probability) (**Figure 7A**). Nevertheless, SKL and PRL are both canonical PTS1 signals found in most eukaryotes that have been shown to function in plants (Lingner et al., 2011; Reumann et al., 2012). PredPlantPTS1 takes into account not only the *C*-terminal tripeptide but also the preceding 11 amino acids, and the negative prediction for ChEC96 reflects the presence of residues that potentially inhibit peroxisome targeting (Reumann et al., 2012). However, ChEC96 was predicted to contain a PTS1 signal (56.7% targeting probability) by a fungi-specific prediction model (Nötzel et al., 2016). The protein sequence of ChEC89, which is less than half the length of ChEC51a and ChEC96 (**Figure 7A**), was not predicted to contain either PTS1 or PTS2 signals using PredPlantPTS1, PTS1Prowler (Bodén and Hawkins, 2005) or PeroxisomeDB (Schlüter et al., 2010).

To experimentally verify the functionality of the *C*-terminal tripeptides in ChEC51a and ChEC96, we deleted the corresponding nucleotide sequences and added an artificial stop codon. The transient expression of these truncated proteins showed that they were no longer targeted to plant peroxisomes and instead showed a uniform nucleo-cytoplasmic distribution (**Figure 7B**). Based on these experiments, we conclude that both ChEC51a and ChEC96 harbor functional PTS1 tripeptides that are required to permit their translocation into plant peroxisomes *via* the classical PTS1 pathway.

### ChECs Labeling Plant Microtubules

**FIGURE 8:**
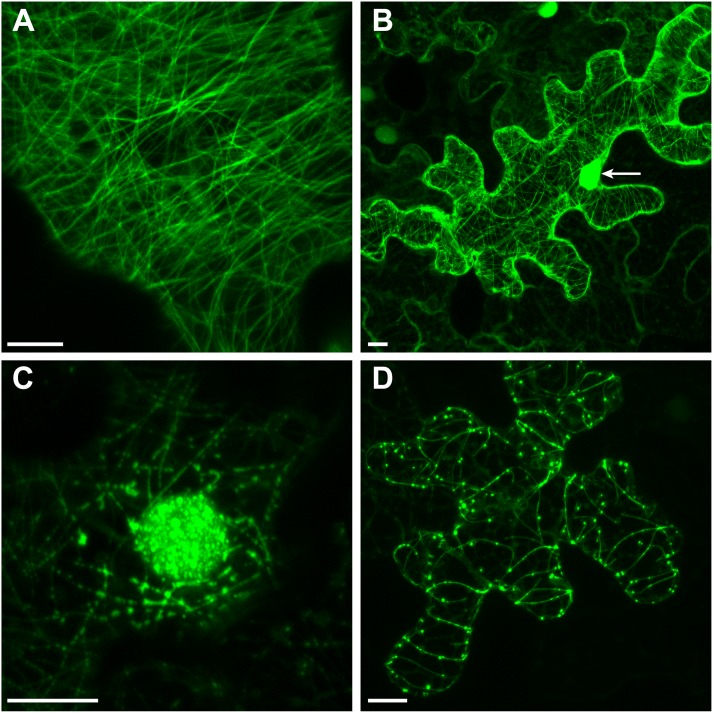
GFP-tagged ChECs decorating plant microtubules upon transient expression in *N. benthamiana* epidermal cells. **(A)** Z-stack projection showing ChEC9 labeling plant microtubules. **(B)** Z-stack projection showing ChEC17 labeling plant microtubules and nucleus (arrow). **(C)** Single optical section showing ChEC17 labeling punctate structures inside a plant nucleus and along microtubules. **(D)** Z-stack projection of a cell expressing GFP-ChEC113, showing labeling of plant microtubules and small punctae closely associated with microtubules. Bar = 10 μm.

Three effector candidates, namely ChEC9, ChEC17, and ChEC113, labeled arrays of long filamentous structures in the cortical cytoplasm of *N. benthamiana* cells which resembled elements of the plant cytoskeleton (**Figure 8**). GFP-ChEC9 exclusively labeled these cytoskeleton filaments (**Figure 8A**) whereas GFP-ChEC17 was also concentrated in granular, punctate structures located along the filaments and inside plant nuclei (**Figures 8B,C**). However, no NLS was detected in the protein sequence of ChEC17 using NLSpredict, cNLS Mapper, NLStradamus, or WoLF PSORT. Similar to GFP-ChEC9, GFP-ChEC113 also labeled small punctate structures that were closely associated with the cytoskeleton filaments, but nuclei were not labeled by this fusion protein (**Figure 8D**).

**FIGURE 9:**
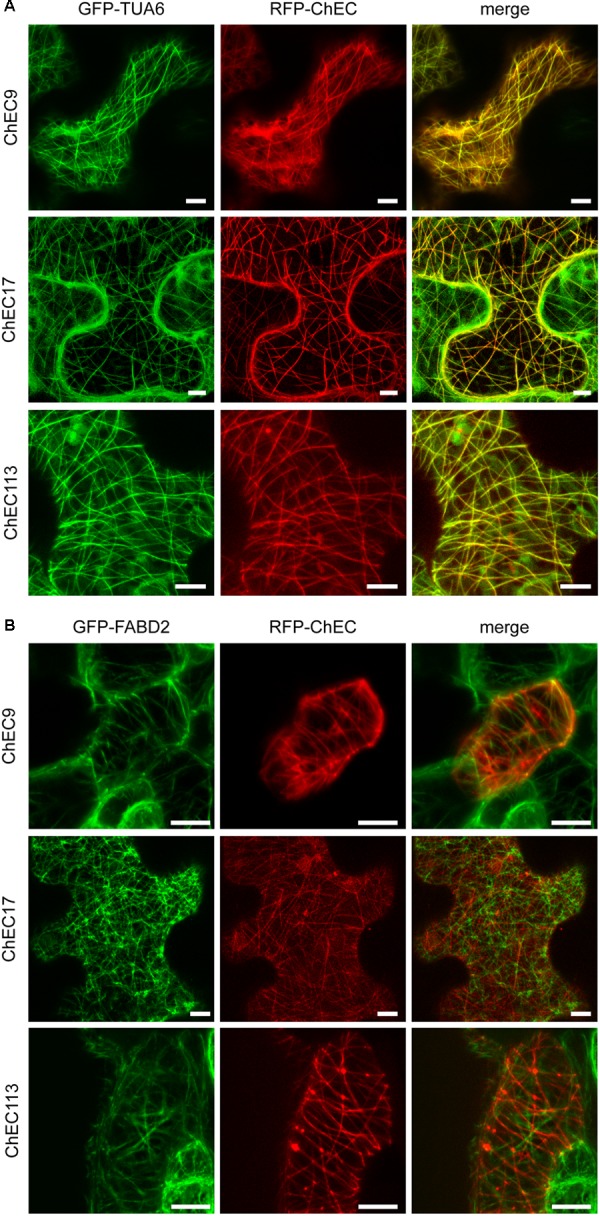
**(A)** Representative confocal microscope z-stack projections showing that RFP-labeled ChEC9, ChEC17, and ChEC113 colocalize with plant cortical microtubule arrays labeled by GFP-TUA6 (plant α-tubulin). Bars = 7.5 μm. **(B)** Confocal microscope z-stack projections showing that RFP-labeled ChEC9, ChEC17, and ChEC113 do not co-localize with plant actin filaments labeled by GFP-FABD2 (fimbrin actin-binding domain 2). Bars = 10 μm.

In order to distinguish whether these proteins were associated with plant microtubules or actin microfilaments, we either transiently expressed them as *N*-terminal RFP fusions in transgenic *N. benthamiana* plants (line CB13) stably expressing tubulin alpha 6 (TUA6) fused to GFP (Ueda et al., 1999; Gillespie et al., 2002), or transiently co-expressed them as *N*-terminal RFP fusions together with the fimbrin actin-binding domain 2 (FABD2) fused to GFP (Sheahan et al., 2004; Voigt et al., 2005). In these experiments, we found that ChEC9, ChEC17, and ChEC113 were all perfectly co-localized with GFP-TUA6-labeled microtubules in every *N. benthamiana* cell examined (**Figure 9A**). In contrast, the RFP-tagged ChECs displayed no detectable co-localization with actin microfilaments (**Figure 9B**). Consistent with this microtubule localization, ChEC113 was predicted to be a microtubule-associated protein using the MAPanalyzer tool (Zhou et al., 2015); however, ChEC9 and ChEC17 were not.

## Discussion

In this study, we analyzed the subcellular localization of 61 biotrophy-expressed effector candidates from *C. higginsianum* using a medium-throughput screen based on their heterologous expression as FP fusions in *N. benthamiana* leaves. The majority of the proteins (40) showed a nucleo-cytoplasmic distribution similar to free GFP. However, it should be noted that all the fusion proteins were tagged at their *N*-termini, which in some cases could have masked other *N*-terminal motifs required for their correct subcellular targeting. Among the 16 ChECs that could be assigned specific localizations, more than half were imported into plant nuclei while the remainder were addressed to compartments that were not previously reported for the effectors of other filamentous pathogens, namely plant microtubules, Golgi and peroxisomes. It must be acknowledged that in our approach the subcellular localization of the putative effectors could be affected by the addition of a large protein tag, strong over-expression of the proteins, and expression in a heterologous plant where the appropriate host targets may not be present. Moreover, there is currently no direct evidence that any of these fungal proteins are translocated into host cells during infection. Despite these caveats, the fact that some ChECs were targeted to specific plant compartments is consistent with the view that these are *bona fide* cytoplasmic effectors, and as such they have high priority for functional analysis. For that purpose, knowledge of their subcellular localizations may assist the future identification of potential host interacting proteins.

We found that nine ChECs (14.5% of those screened) localized entirely to plant nuclei upon transient expression in either *N. benthamiana* or *A. thaliana*, the natural host plant. All of these proteins carry a predicted NLS motif and are therefore likely to be actively translocated through nuclear pore complexes by means of the plant nuclear import machinery (Wirthmueller et al., 2013). In the maize anthracnose pathogen, *C. graminicola*, an *in silico* screen identified 27 putative nuclear-targeted effectors that contain both a secretion signal and an NLS (Vargas et al., 2016). One of these, CgEP1, was verified to accumulate in maize nuclei during infection and was shown to bind to the promoters of several maize genes, potentially regulating their expression. In a subcellular localization screen similar to the one used here, Caillaud and co-workers (2012) likewise found that among 49 effectors from the oomycete *Hyaloperonospora arabidopsidis*, the nucleus was the most frequently targeted host cell compartment, with 16 (33%) of the proteins showing an exclusively nuclear localization. Numerous effectors from other oomycetes, fungi, bacteria, and nematodes have also been shown to translocate into plant nuclei (Kemen et al., 2005; Boch and Bonas, 2010; Schornack et al., 2010; Rivas and Genin, 2011; Weßling et al., 2014; Vargas et al., 2016; Boevink et al., 2016), indicating that the manipulation of plant nuclear processes is a virulence strategy shared by pathogens from diverse kingdoms of life.

The nine nuclear-targeted ChECs labeled distinct nuclear compartments. For example, ChEC74, ChEC98, ChEC104, ChEC106, ChEC108, and ChEC111 labeled the nucleoplasm but accumulated preferentially in the nucleolus, where they could potentially manipulate major nucleolar functions such as the transcription and processing of ribosomal RNA and ribosome assembly (Shaw and Brown, 2012). In addition to the nucleolus, ChEC108 also accumulated in a sub-set of Cajal bodies labeled by the markers FIB2 and U2B. Cajal bodies harbor diverse functions that partially overlap with those of the nucleolus, including RNA metabolism, gene silencing and the formation of ribonucleoprotein particles involved in transcription, pre-mRNA splicing, ribosome biogenesis and telomere maintenance (Love et al., 2017). Three other nuclear-targeted ChECs were excluded from the nucleolus and remained within the nucleoplasm, and one of these, ChEC118, altered the distribution of nuclear DNA in *N. benthamiana*, with the fungal protein accumulating in areas of the nucleoplasm from which DNA was depleted. This suggests that ChEC118 may induce the reorganization of plant chromatin structure, similar to the *Phytophthora capsici* effector CRN83_152 (Stam et al., 2013).

One striking finding was that the transient over-expression of GFP-ChEC106 in *N. benthamiana* cells caused a nearly 3-fold increase in the area of plant nuclei. Given that nuclear size is known to correlate well with ploidy level in plants (Bourdon et al., 2011), our finding raises the possibility that ChEC106 increases the ploidy level of infected host cells. Interestingly, the invasion of *A. thaliana* leaf epidermal cells by haustoria of the powdery mildew fungus *Golovinomyces orontii* is associated with an increase in nuclear volume and ploidy level of the underlying mesophyll cells due to endoreduplication (Chandran et al., 2010), and localized host endoreduplication is induced by several fungal and bacterial symbionts (Wildermuth, 2010). However, we detected no obvious inflation of transformed nuclei upon transient expression of GFP-ChEC106 in *A. thaliana*, and further work is now required to determine whether the ploidy of host cells is altered during biotrophic infection by *C. higginsianum*.

Our finding that three candidate effectors, ChEC9, ChEC17, and ChEC113, label plant microtubules suggests that subversion of microtubule-dependent plant functions may be important for the successful invasion of host cells by *C. higginsianum*. There is increasing evidence that microtubule networks contribute to plant immunity. For example, upon attack by non-adapted pathogens, and unsuccessful penetration attempts by adapted pathogens, plant cortical microtubules undergo reorganization into radial patterns focused on fungal entry sites (Hoefle et al., 2011). Moreover, treatment with microtubule inhibitors such as oryzalin weakens resistance to both bacteria and fungi (Kobayashi et al., 1997; Lee et al., 2012). There are no previous reports of fungal or oomycete effectors interacting with plant microtubules. However, two type III effectors from *Pseudomonas syringae*, HopZ1a and HopE1, are known to promote bacterial virulence by targeting microtubules. HopZ1a is a plasma membrane-localized acetyltransferase that destroys host cortical microtubule arrays (Lee et al., 2012). Consistent with the role of microtubules in vesicle trafficking and polarized secretion (Crowell et al., 2009), HopZ1a inhibited protein secretion in *N. benthamiana* and blocked PAMP-induced callose deposition in *Arabidopsis* (Guo et al., 2016). HopE1 causes the microtubule cross-linking protein MAP65 to dissociate from microtubules without disrupting cortical arrays, but similar to HopZ1a, plants expressing HopE1 are impaired in the secretion of immunity-related proteins and callose deposition (Guo et al., 2016). In future work, it will be important to determine if these three ChECs interact directly with tubulin or with microtubule-associated proteins, and what impact they have on microtubule dynamics and plant immune responses.

Colocalization with specific plant organelle markers allowed us to identify plant Golgi and peroxisomes as the destinations for four ChECs. Thus, we found that ChEC21 specifically labels plant Golgi stacks, which raises the possibility that ChEC21 functions to manipulate host vesicle trafficking. The bacterial type III effector XopJ from *Xanthomonas campestris* pv. *vesicatoria* also localizes to plant Golgi and inhibits plant secretion and callose deposition (Bartetzko et al., 2009). The *P. syringae* pv. *tomato* effector HopM1 likewise blocks secretion-mediated immunity, in this case by targeting a key regulator of vesicle trafficking for proteasome degradation, namely the *Arabidopsis* ADP ribosylation factor guanine nucleotide exchange factor (ARF-GEF) AtMIN7 (Nomura et al., 2011). However, unlike ChEC21 and XopJ, which both associate with plant Golgi, HopM1 localizes to the *trans*-Golgi network/early endosome compartment. It is interesting to note that several genera of ascomycete fungal pathogens and endophytes have also evolved to subvert plant vesicle trafficking and secretion by producing the macrolide metabolite brefeldin A and related molecules that inhibit ARF-GEF function, similar to HopM1 (Kwon et al., 2008).

Remarkably, out of the 61 effector candidates screened in our study, no fewer than three, ChEC51a, ChEC89, and ChEC96, were specifically targeted to plant peroxisomes when expressed in *N. benthamiana* as *N*-terminal fusions with either GFP or RFP. The corresponding fungal genes show identical expression profiles, being upregulated specifically in the biotrophic phase of infection, and ChEC89 and ChEC96 both rank among the 60 most highly expressed fungal genes during biotrophy (**Figure 1B**; O’Connell et al., 2012). These findings suggest that the manipulation of host peroxisome activities may be especially important during the early colonization of living host cells by *C. higginsianum*. Peroxisomes harbor diverse metabolic processes that contribute to plant immunity. These include jasmonic acid biosynthesis, the generation of reactive oxygen species (H_2_O_2_, superoxide radicals, NO) and, in *Arabidopsis*, the enzymatic activation of antimicrobial indole glucosinolates, which restrict the growth of many fungal pathogens, including *Colletotrichum* (Lipka et al., 2005; Clay et al., 2009; Kaur et al., 2009; Hiruma et al., 2010).

Previously we found that when ChEC89-RFP was expressed in *C. higginsianum* the fusion protein was secreted to the surface of biotrophic hyphae and accumulated in interfacial bodies, but no fluorescence was detectable in the host cytoplasm (Kleemann et al., 2012). However, when delivered directly into the cytoplasm of *Arabidopsis* cells *via* the bacterial type 3 secretion system, ChEC89 enhanced bacterial virulence, presumably by suppressing host immune responses (Kleemann et al., 2012). Together with our present finding that ChEC89 is specifically imported into peroxisomes, this supports the view that during infection this protein is translocated across the plasma membrane to function inside host cells.

In all eukaryotes, proteins destined for import into the peroxisome matrix are first recognized in the cytosol by soluble receptors that guide them to specific docking sites on the peroxisome membrane for translocation into the peroxisome matrix (Platta and Erdmann, 2007). Here, we show that both ChEC51a and ChEC96 contain functional PTS1 signals, SKL and PRL, respectively, that are essential for their translocation into plant peroxisomes *via* the PTS1 pathway. SKL and PRL are canonical PTS1 tripeptides that are highly represented among the peroxisome matrix proteins of higher plants (Reumann, 2004; Lingner et al., 2011). However, neither PTS1 nor PTS2 signals were detectable in the protein sequence of ChEC89 using available prediction algorithms. We therefore speculate that ChEC89 could be imported into the peroxisomal matrix by associating with plant proteins that harbor appropriate targeting signals.

Using confocal microscopy, GFP-ChEC89 was evenly distributed through the peroxisomal matrix, whereas GFP-ChEC51a and GFP-ChEC96 were concentrated in smaller punctate structures within the matrix. Catalase crystals are the only inclusions known to occur in plant peroxisomes (Frederick and Newcomb, 1969). With the higher resolution of immunogold-TEM, GFP-ChEC96 could be found concentrated in small electron-opaque inclusions, but in those sections where the rhomboid catalase crystal was visible, no labeling of the crystal was detectable with anti-GFP antibodies. Thus, the identity of the punctate inclusions labeled by GFP-ChEC51a and GFP-ChEC96 remains to be determined.

To our knowledge, no effector proteins from other fungal, oomycete or bacterial pathogens have been reported to associate with plant peroxisomes. However, in a cell biology screen similar to that used here, two effectors from the potato cyst nematode *Globodera pallida* associated with peroxisomes when transiently expressed in *N. benthamiana*, one localizing to the peroxisome matrix and the other labeling peroxisomal membranes (Thorpe et al., 2014). Taken together with our data, this suggests that plant pathogens belonging to two different eukaryotic kingdoms have evolved secreted effectors to manipulate the activities of plant peroxisomes. ChEC51a, ChEC89, and ChEC96 contain no recognizable protein domains that might give clues to their functions. Work is on-going to identify plant proteins interacting with these ChECs in order to elucidate their biological functions and molecular targets.

## Author Contributions

GR and RO: conceived and designed the experiments and wrote and revised the manuscript. GR, UN, LC, and RO: performed the cell biology experiments and interpreted the data. JK, J-FD, and NL: performed the computational analyses and interpreted the data. GR, UN, J-FD, and LC: prepared the figures and tables. All authors helped to edit the manuscript and approved the final version.

## Conflict of Interest Statement

The authors declare that the research was conducted in the absence of any commercial or financial relationships that could be construed as a potential conflict of interest.
