# Supplementary material for: Subcellular Localization Screening of Colletotrichum higginsianum Effector Candidates Identifies Fungal Proteins Targeted to Plant Peroxisomes, Golgi Bodies, and Microtubules
Source: Front Plant Sci. 2018 May 2;9:562. doi: 10.3389/fpls.2018.00562 (PMC5942036; doi:10.3389/fpls.2018.00562)
Supplement: Supplementary file 1 [file Table_1.PDF]

Supplementary Table 1: Properties of selected *Colletotrichum higginisianum* effector candidates

Protein ID	Gene ID	No. amino acids (a)	Size (kDa) (b)	No. Cys	Expression stage (c)	Localization (d)	LOCALIZER prediction (e)	WoLF PSORT prediction (f)
ChEC3	CH63R_13445	70 (23)	5.6	2	PA + BP	Nucleocytoplasmic		Cytoplasmic
ChEC4	CH63R_09505	107 (16)	9.63	0	PA + BP	Nucleus	Nucleus	
ChEC6	CH63R_13188	89 (18)	7.95	0	PA + BP	Nucleocytoplasmic		Cytoplasmic, nuclear
ChEC7	CH63R_14383	85 (16)	7.19	8	PA	Nucleocytoplasmic		Chloroplast
ChEC8	CH63R_03716	120 (18)	11.8	1	PA + BP	Nucleocytoplasmic	Nucleus	Nuclear
ChEC9	CH63R_02052	301 (18)	32.07	0	PA	Microtubules	Nucleus	Peroxisomal
ChEC11	unitig 10 (1208247-1208455)	47 (19)	3.1	6	PA + BP	Nucleocytoplasmic		
ChEC12	CH63R_14516	97 (15)	9.25	6	BP	Nucleus		Chloroplast, mitochondrial
ChEC14	CH63R_03968	103 (18)	9.56	8	PA + BP	Nucleocytoplasmic	Nucleus	Chloroplast
ChEC15	unitig 5 (245377-245562)	61 (19)	4.79	2	PA + BP	Nucleocytoplasmic		Cytoplasmic
ChEC16	CH63R_08782	82 (20)	8.63	5	BP	Nucleocytoplasmic		Mitochondrial
ChEC17	CH63R_12467	64 (19)	12.94	4	PA + BP	Nucleus, microtubules		Chloroplast
ChEC19	CH63R_13308	122 (22)	10.86	0	PA + BP	Nucleocytoplasmic		Cytoplasmic
ChEC20	CH63R_01410	109 (17)	10.07	6	BP	Nucleocytoplasmic		Chloroplast
ChEC21	CH63R_14507	143 (24)	13.21	8	PA + BP	Golgi		Nuclear
ChEC21a	unitig 6 (3993669-3994290)	114 (25)	10.38	0	PA + BP	Cytosol		
ChEC22	unitig 6 (4099234-4099575)	114 (18)	12.01	6	BP	Nucleocytoplasmic		Chloroplast
ChEC27	CH63R_14267	46 (19)	6.76	2	PA + BP	Nucleocytoplasmic		Extracellular
ChEC28	unitig 6 (4190825-4191025)	310 (22)	4.75	10	PA + BP	Nucleocytoplasmic		Chloroplast
ChEC30	CH63R_02802	96 (18)	16.78	4	PA + BP	Cytosol		Nuclear
ChEC31	unitig 1 (4882681-4882943)	46 (17)	3.15	2	PA + BP	Nucleocytoplasmic		
ChEC32	CH63R_05615	202 (21)	21.4	0	BP	Nucleocytoplasmic	Nucleus	Cytoplasmic
ChEC34	CH63R_11505	85 (19)	7.14	7	BP	Nucleocytoplasmic		Nuclear
ChEC36	CH63R_14377	225 (13)	16.18	6	PA + BP	Small organelles	Chloroplast	
ChEC39	CH63R_12542	163 (18)	16.2	6	BP	Small organelles		
ChEC41	CH63R_09582	127 (26)	10.6	3	BP	Nucleocytoplasmic		Chloroplast
ChEC45	CH63R_12164	118 (18)	9.79	2	BP	Nucleocytoplasmic	Nucleus	
ChEC50	unitig 10 (780674-780835)	53 (17)	4.02	1	PA + BP	Nucleocytoplasmic		Cytoplasmic, extracellular
ChEC51	CH63R_10425	108 (25)	24.62	0	BP	Nucleocytoplasmic	Nucleus	Nuclear
ChEC51a	CH63R_14648 (g)	197 (16)	20.55	0	BP	Peroxisomes		Peroxisomal
ChEC73	unitig 1 (3922414-3922557)	47 (24)	2.89	2	PA + BP	Nucleocytoplasmic		
ChEC74	CH63R_08130	335 (18)	31.1	1	PA + BP	Nucleus	Nucleus	Nuclear
ChEC79	unitig 4 (4368041-4368184)	47 (26)	2.72	0	PA + BP	Nucleocytoplasmic		
ChEC85	CH63R_03836	86 (17)	7.18	5	BP	Nucleocytoplasmic		Chloroplast
ChEC87	CH63R_05074	64 (17)	5.11	6	BP	Nucleocytoplasmic		Cytoplasmic
ChEC89	CH63R_05511	83 (19)	6.8	4	BP	Peroxisomes		Chloroplast
ChEC92	CH63R_01492	62 (19)	4.97	0	PA + BP	Nucleocytoplasmic		Cytoplasmic
ChEC93	CH63R_02466	94 (21)	8.14	10	PA + BP	Nucleocytoplasmic		Chloroplast
ChEC94	CH63R_03399	67 (18)	4.89	7	PA + BP	Nucleocytoplasmic		Chloroplast
ChEC95	CH63R_12106	77 (19)	6.58	8	BP	Nucleocytoplasmic		Extracellular
ChEC96	CH63R_05575	181 (23)	17.45	5	BP	Peroxisomes	Nucleus	Nuclear
ChEC97	CH63R_05409	123 (16)	11.96	0	PA + BP	Nucleocytoplasmic		
ChEC98	CH63R_12158	224 (21)	20.59	4	PA + BP	Nucleus, aggregates	Nucleus	Nuclear
ChEC99	CH63R_10618	190 (18)	20.04	4	BP	Nucleocytoplasmic	Nucleus	
ChEC100	CH63R_00329	77 (18)	6.38	8	BP	Nucleocytoplasmic		Extracellular
ChEC101	CH63R_06128	93 (17)	7.34	6	BP	Nucleocytoplasmic		Chloroplast
ChEC103	CH63R_06491	89 (22)	6.99	10	PA + BP	Cytosol		
ChEC104	CH63R_01904	180 (21)	17.71	2	BP	Nucleus	Nucleus	Cytoplasmic
ChEC105	CH63R_05052	84 (17)	7.02	4	BP	Nucleocytoplasmic		
ChEC106	CH63R_00096	153 (24)	14.47	6	PA + BP	Nucleus	Nucleus	Nuclear
ChEC107	CH63R_02213	142 (20)	13.1	10	PA + BP	Nucleocytoplasmic		Chloroplast
ChEC108	CH63R_09563	216 (20)	22.96	3	BP	Nucleus	Nucleus	Nuclear
ChEC109	CH63R_05391	158 (19)	15.4	0	PA	Nucleocytoplasmic		Cytoplasmic
ChEC110	CH63R_02800	117 (34)	9.35	6	PA + BP	Nucleocytoplasmic		Chloroplast
ChEC111	CH63R_03265	107 (24)	9.12	2	BP	Nucleus	Nucleus	Nuclear
ChEC112	CH63R_01482	60 (23)	6.34	2	BP	Nucleocytoplasmic		
ChEC113	CH63R_03987	411 (18)	43.52	0	PA + BP	Microtubules	Nucleus	Nuclear
ChEC114	CH63R_09752	68 (16)	5.45	7	PA + BP	Nucleocytoplasmic		Chloroplast
ChEC116	CH63R_14384	73 (16)	6.78	6	BP	Nucleocytoplasmic		
ChEC117	unitig 6 (4027978-4028255)	76 (19)	6.65	2	BP	Nucleocytoplasmic		
ChEC118	CH63R_03498	66 (20)	5.35	0	PA + BP	Nucleus	Nucleus	Nuclear

(a) Mature protein length (signal peptide length given in parentheses).
(b) Predicted size of mature protein (without signal peptide).
(c) Preferential gene expression in appressoria *in planta* (PA) and/or biotrophic phase (BP), based on RNA-seq transcript profiling (O'Connell et al. 2012).
(d) Localization determined in this study.
(e) Localization predicted using LOCALIZER in 'effector' mode (Sperschneider et al. 2017).
(f) Localization predicted using WoLF PSORT in 'plant' mode (Horton et al. 2007). The localizations shown are those found in at least 5 proteins out of 14 reference proteins reported by WoLF PSORT. If no localization passed this threshold, we considered the result too noisy to present.
(g) Gene model is C-terminally truncated.
