# Supplementary material for: Subcellular Localization Screening of Colletotrichum higginsianum Effector Candidates Identifies Fungal Proteins Targeted to Plant Peroxisomes, Golgi Bodies, and Microtubules
Source: Front Plant Sci. 2018 May 2;9:562. doi: 10.3389/fpls.2018.00562 (PMC5942036; doi:10.3389/fpls.2018.00562)
Supplement: Supplementary file 2 [file Table_2.PDF]

Supplementary Table 2: Presence of proteins homologous to *C. higginsianum* effector candidates inside and outside the genus *Colletotrichum*

Category of effector	ChEC	*Colletotrichum* species having homologous proteins (a)	Other fungi having homologous protein(s)
C. higginsianum "species-specific" (20)	ChEC12	-	-
	ChEC15	-	-
	ChEC19	-	-
	ChEC21	-	-
	ChEC21a	-	-
	ChEC31	-	-
	ChEC39	-	-
	ChEC45	-	-
	ChEC73	-	-
	ChEC74	-	-
	ChEC79	-	-
	ChEC94	-	-
	ChEC98	-	-
	ChEC108	-	-
	ChEC109	-	-
	ChEC111	-	-
	ChEC112	-	-
	ChEC113	-	-
	ChEC116	-	-
	ChEC117	-	-
Colletotrichum "genus-specific" (30)	ChEC3	*C.glo,C.orb*	-
	ChEC4	*C.gra*	-
	ChEC7	*C.chl,C.fio,C.gra,C.inc,C.nym,C.orc,C.sal,C.sim,C.tof*	-
	ChEC8	*C.chl,C.glo,C.inc,C.nym,C.orb,C.sal,C.sim,C.sub,C.tof*	-
	ChEC9	*C.inc,C.tof*	-
	ChEC11	*C.glo*	-
	ChEC16	*C.fio,C.glo,C.inc,C.nym,C.sal,C.sim*	-
	ChEC17	*C.chl,C.fio,C.glo,C.inc,C.nym,C.orb,C.orc,C.sal,C.sim*	-
	ChEC20	*C.chl,C.fio,C.glo,C.inc,C.nym,C.orc,C.sal,C.sim,C.tof*	-
	ChEC22	*C.fio,C.glo,C.orb,C.orc,C.sal,C.sim*	-
	ChEC28	*C.glo,C.orb*	-
	ChEC30	*C.sal*	-
	ChEC34	*C.chl,C.fio,C.glo,C.inc,C.nym,C.orb,C.sal,C.tof*	-
	ChEC41	*C.gra,C.sub*	-
	ChEC50	*C.tof*	-
	ChEC51	*C.orb,C.sal,C.sub*	-
	ChEC85	*C.orb*	-
	ChEC87	*C.chl,C.glo,C.gra,C.inc,C.orc,C.tof*	-
	ChEC92	*C.chl,C.gra,C.inc,C.orc*	-
	ChEC95	*C.fio,C.inc,C.sal*	-
	ChEC96	*C.glo,C.orb*	-
	ChEC97	*C.fio,C.orc,C.sal,C.sim,C.tof*	-
	ChEC99	*C.nym,C.orb,C.sal,C.sim*	-
	ChEC100	*C.fio,C.glo,C.inc,C.nym,C.sim*	-
	ChEC103	*C.glo,C.nym,C.sim,C.sub*	-
	ChEC104	*C.orb*	-
	ChEC106	*C.glo,C.gra,C.inc,C.orc,C.sal,C.sub*	-
	ChEC110	*C.chl,C.fio,C.glo,C.inc,C.nym,C.orc,C.sal,C.sim,C.tof*	-
	ChEC114	*C.chl,C.nym,C.sal*	-
	ChEC118	*C.chl,C.fio,C.glo,C.gra,C.inc,C.nym,C.orb,C.orc,C.sal,C.sim, C.tof*	-
"Non-specific" (11)	ChEC6	*C.glo,C.gra,C.orb*	*Penicillium vulpinum*
	ChEC14	*C.fio,C.glo,C.nym,C.orc,C.sal,C.sim*	*Fusarium oxysporum f. sp*
	ChEC27	*C.nym,C.orb,C.orc,C.sal,C.sim*	*Diaporthe ampelina, Diaporthe helianthi, Diplocarpon rosae, Rhynchosporium agropyri, Rhynchosporium commune*
	ChEC32	*C.glo,C.nym,C.orb,C.orc,C.sim,C.sub*	*Diaporthe helianthi*
	ChEC36	*C.chl,C.fio,C.inc,C.nym,C.orb,C.sal,C.sim*	*Fusarium oxysporum sp*
	ChEC51a	*C.fio,C.nym,C.orb,C.orc,C.sal,C.sim,C.sub*	*Ceratocystis fimbriata*
	ChEC89	*C.fio,C.glo,C.nym,C.orb,C.sal,C.sim*	*Diaporthe ampelina*
	ChEC93	*C.chl,C.fio,C.glo,C.inc,C.nym,C.orb,C.sal,C.sim,C.tof*	*Diplodia corticola*
	ChEC101	*C.chl,C.fio,C.glo,C.inc,C.nym,C.orb,C.orc,C.sal,C.sim,C.tof*	*Pseudomassariella vexata*
	ChEC105	*C.fio,C.glo,C.nym,C.orb,C.sal,C.sim*	*Diaporthe ampelina*
	ChEC107	*C.gra,C.inc,C.orb,C.tof*	*Dichomitus squalens, Magnaporthe oryzae, Microdochium bolleyi*

(a) Species abbreviations used :

C. gloeosporioides	*C.glo*
C. orbiculare	*C.orb*
C. orchidophilum	*C.orc*
C. incanum	*C.inc*
C. simmondsii	*C.sim*
C. fioriniae	*C.fio*
C. nymphaeae	*C.nym*
C. sublineola	*C.sub*
C. graminicola	*C.gra*
C. salicis	*C.sal*
C. chlorophyti	*C.chl*
C. tofieldiae	*C.tof*
