# Supplementary material for: Subcellular Localization Screening of Colletotrichum higginsianum Effector Candidates Identifies Fungal Proteins Targeted to Plant Peroxisomes, Golgi Bodies, and Microtubules
Source: Front Plant Sci. 2018 May 2;9:562. doi: 10.3389/fpls.2018.00562 (PMC5942036; doi:10.3389/fpls.2018.00562)
Supplement: Supplementary file 3 [file Table_3.PDF]

Supplementary Table 3: Predicted localizations for nine nuclear-targeted effectors of *Colletotrichum higginsianum*

		Prediction algorithm					
**Effector**	**Gene ID**	**Predict NLS[(a)]**	**cNLS Mapper[(b)]**	**NLS tradamus [(c)]**	**WoLFP SORT[(d)]**	**Nucleolus [(e)]**	**Experimentally determined localization [(f)]**
ChEC4	CH63R_09505	+	(3.9)	+	+	-	Nucleoplasm
ChEC12	CH63R_14516	-	(5.8)	-	-	+	Nucleoplasm
ChEC74	CH63R_08130	+	(3.8)	+	+	+	Nucleolus > nucleoplasm
ChEC98	CH63R_12158	+	0	+	+	-	Nucleolus > nucleoplasm
ChEC104	CH63R_01904	+	Bip (7.6) Mon (7.5)	+	-	+	Nucleolus > nucleoplasm
ChEC106	CH63R_00096	-	(3.3)	+	+	+	Nucleolus > nucleoplasm
ChEC108	CH63R_09563	-	(3.9)	+	+	-	Nucleolus + Cajal Bodies
ChEC111	CH63R_03265	+	Bip (6.3)	+	+	+	Nucleolus > nucleoplasm
ChEC118	CH63R_03498	-	Mon (7)	-	+	-	Nucleoplasm

(a) PredictNLS: https://rostlab.org/owiki/index.php/PredictNLS

(b) cNLS MAPPER: http://nls-mapper.iab.keio.ac.jp/cgi-bin/NLS_Mapper_form.cgi. A reliable score is 6 to 7, Mon=monopartite ; Bip=Bipartite ;

(c) NLStradamus: http://www.moseslab.csb.utoronto.ca/NLStradamus/

(d) WoLFPSORT: https://www.genscript.com/wolf-psort.html (plant prediction).

(e) NOD nucleolar localization sequence detector: http://www.compbio.dundee.ac.uk/www-nod/

(f) Data from this study.
