# Supplementary material for: Subcellular Localization Screening of Colletotrichum higginsianum Effector Candidates Identifies Fungal Proteins Targeted to Plant Peroxisomes, Golgi Bodies, and Microtubules
Source: Front Plant Sci. 2018 May 2;9:562. doi: 10.3389/fpls.2018.00562 (PMC5942036; doi:10.3389/fpls.2018.00562)
Supplement: Supplementary file 4 [file Image_1.PDF]

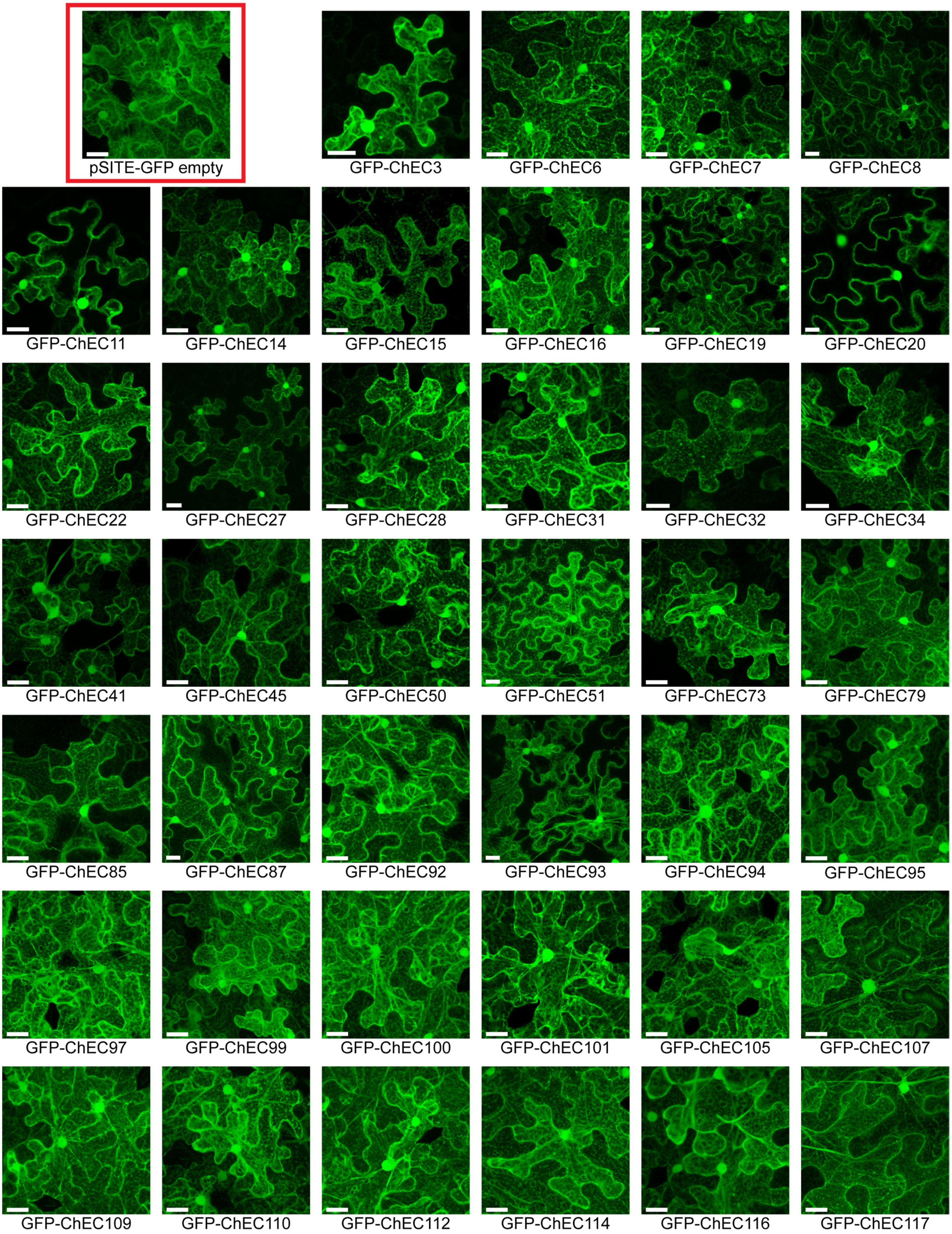

Supplementary Figure 1: Representative confocal microscope z-stack projections showing the nucleo-cytoplasmic distribution of 40 GFP-ChEC fusion proteins upon transient expression in *N. benthamiana* leaf cells. The localization patternof free GFP expressed alone is presented in the top left corner. Bars = 25µm.
