# Supplementary material for: Subcellular Localization Screening of Colletotrichum higginsianum Effector Candidates Identifies Fungal Proteins Targeted to Plant Peroxisomes, Golgi Bodies, and Microtubules
Source: Front Plant Sci. 2018 May 2;9:562. doi: 10.3389/fpls.2018.00562 (PMC5942036; doi:10.3389/fpls.2018.00562)
Supplement: Supplementary file 6 [file Image_3.PDF]

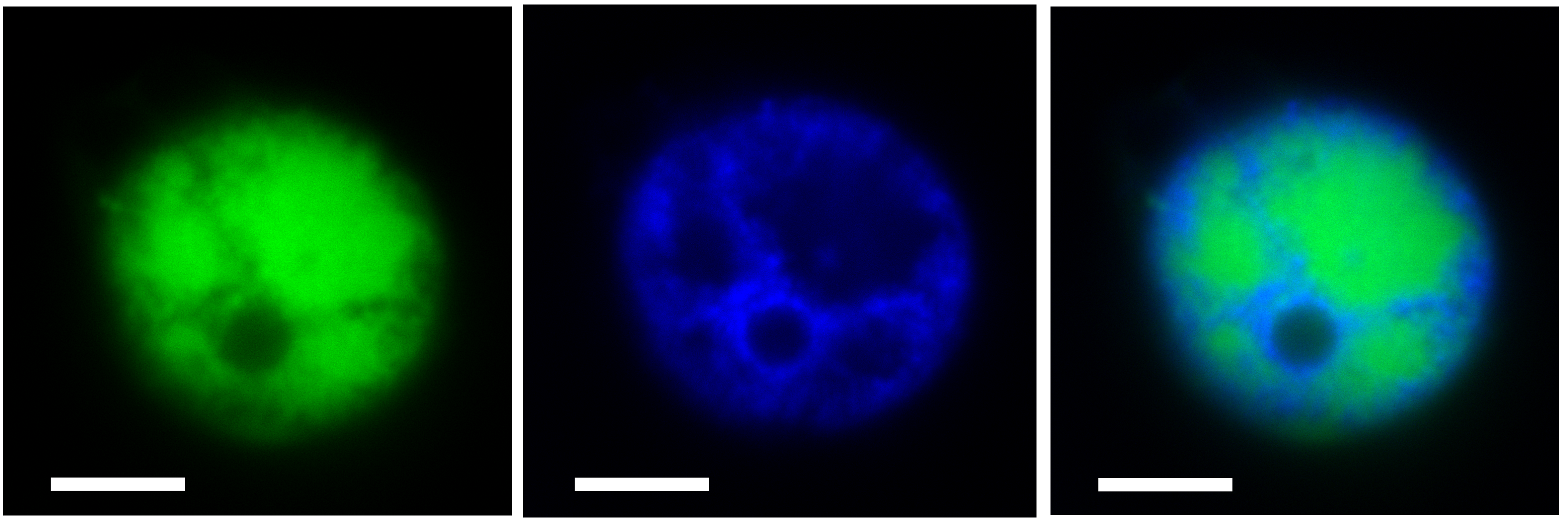

GFP-ChEC118 DAPI merge

Supplementary Figure 3: Confocal microscope single optical section showing that transient expression of GFP-ChEC118 in *N. benthamiana* causes partitioning of plant DNA (stained blue with DAPI) within the nucleoplasm. Bars = 5 μm.
