# Supplementary material for: Subcellular Localization Screening of Colletotrichum higginsianum Effector Candidates Identifies Fungal Proteins Targeted to Plant Peroxisomes, Golgi Bodies, and Microtubules
Source: Front Plant Sci. 2018 May 2;9:562. doi: 10.3389/fpls.2018.00562 (PMC5942036; doi:10.3389/fpls.2018.00562)
Supplement: Supplementary file 7 [file Image_4.PDF]

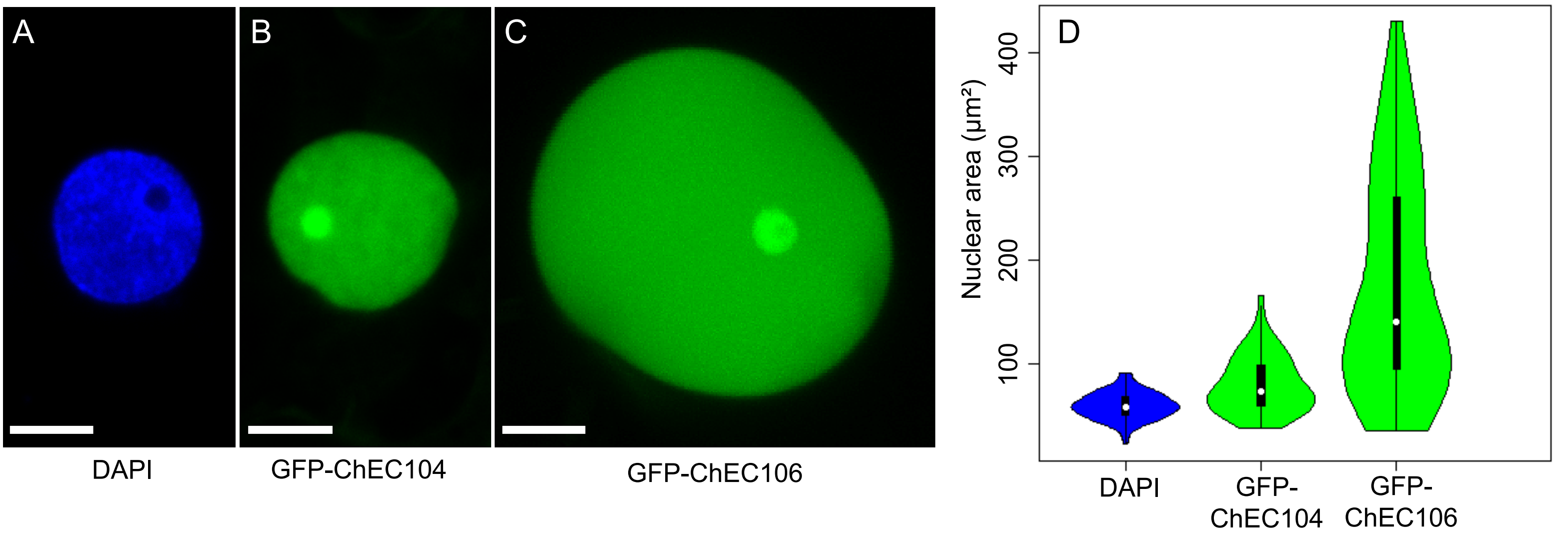

Supplementary Figure 4: Transient expression of GFP-ChEC106 significantly increases the size of *N. benthamiana* nuclei. **(A)** DAPI staining of DNA in an interphase nucleus without transient expression of an effector. **(B)** Nucleus of a cell expressing GFP-ChEC104. **(C)** Nucleus of a cell expressing GFP-ChEC106. Bars = 5 µm. **(D)** Violin plot depicting the frequency distribution of nuclear areas in *N. benthamiana* cells expressing GFP-ChEC106, GFP-ChEC104 or stained with DAPI alone, based on area measurements from 106, 49 and 81 nuclei, respectively. Differences between the three datsets are significant in all pair-wise comparisons (Mann-Whitney non-parametric test, $p< 0.01$).The inner box plots represent the median (white circle), interquartile range (black rectangle) and 95% confidence interval (whiskers).
