# Supplementary material for: Subcellular Localization Screening of Colletotrichum higginsianum Effector Candidates Identifies Fungal Proteins Targeted to Plant Peroxisomes, Golgi Bodies, and Microtubules
Source: Front Plant Sci. 2018 May 2;9:562. doi: 10.3389/fpls.2018.00562 (PMC5942036; doi:10.3389/fpls.2018.00562)
Supplement: Supplementary file 8 [file Image_5.PDF]

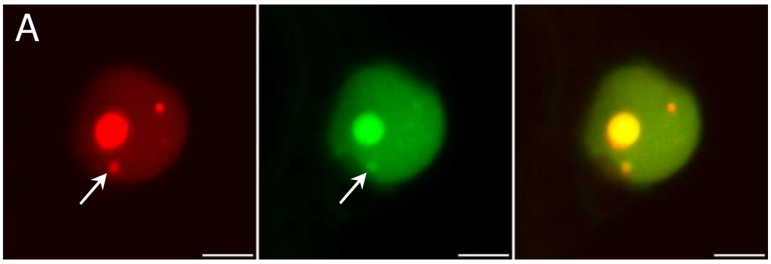

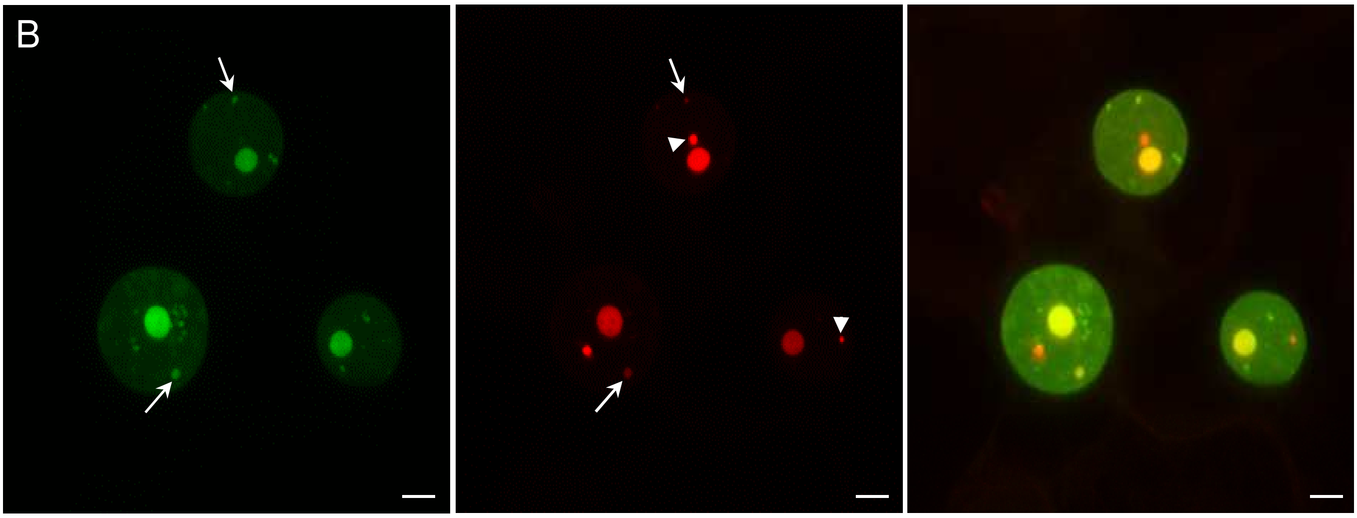

Supplementary Figure 5: (A) Confocal microscope z-stack projections showing part of an *N. Benthamiana* cell co-expressing GFP-tagged ChEC108 and RFP-tagged FIB2 (fibrillarin 2). ChEC108 and FIB2 are co-localized in the nucleolus and Cajal bodies (arrow). **(B)** Confocal microscope z-stack projections showing *N. benthamiana* cells co-expressing RFP-tagged ChEC108 and GFP-tagged U2B (U2 Small nuclear ribonucleoprotein B). ChEC108 and U2B are co-localized in nucleoli and a sub-set of Cajal bodies (arrows). Other sub-nuclear compartments are labelled by ChEC108 only (arrowheads). Bars = 5µm.
