# Supplementary material for: Subcellular Localization Screening of Colletotrichum higginsianum Effector Candidates Identifies Fungal Proteins Targeted to Plant Peroxisomes, Golgi Bodies, and Microtubules
Source: Front Plant Sci. 2018 May 2;9:562. doi: 10.3389/fpls.2018.00562 (PMC5942036; doi:10.3389/fpls.2018.00562)
Supplement: Supplementary file 9 [file Image_6.PDF]

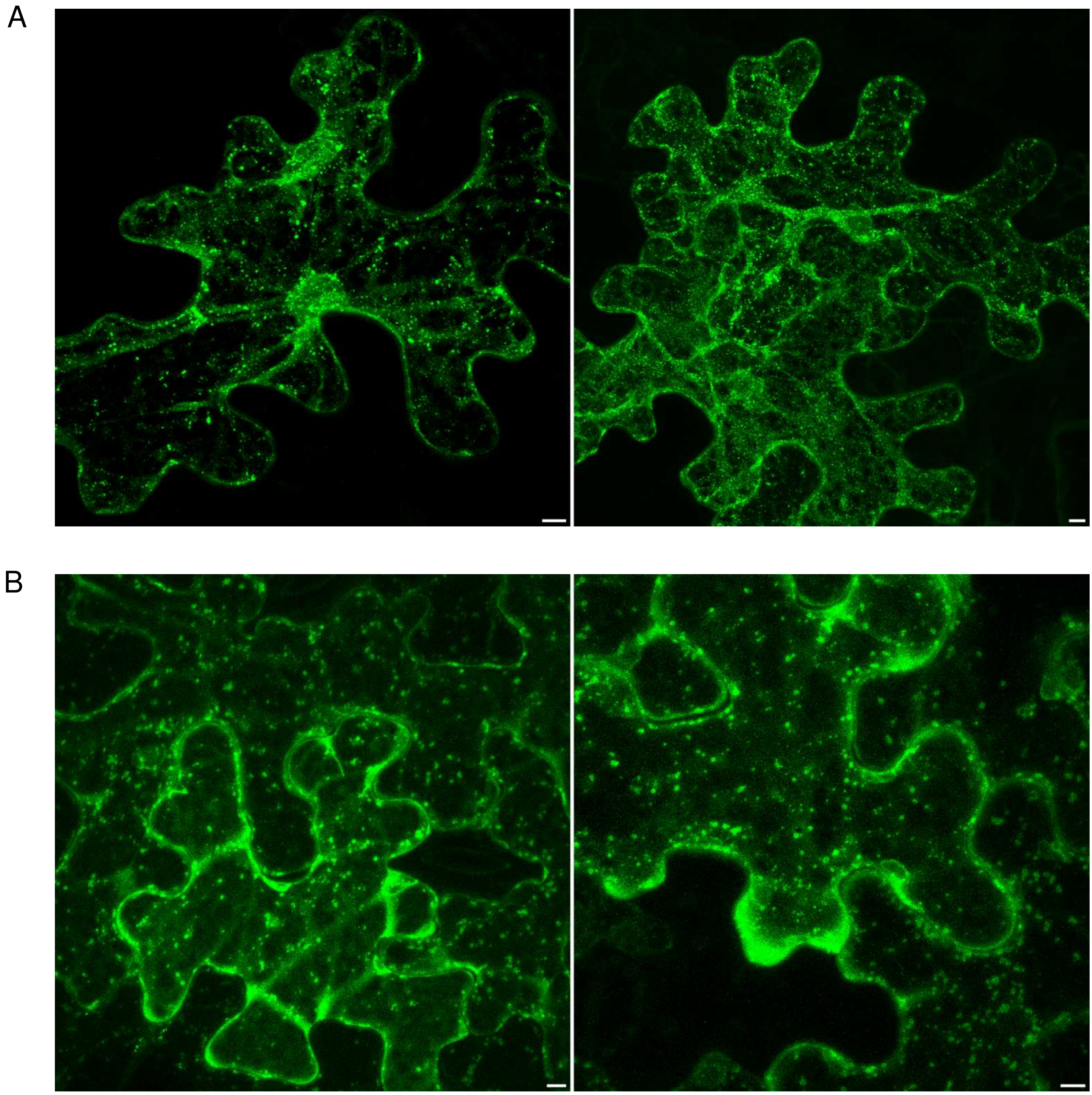

Supplementary Figure 6: Representative confocal microscope z-stack projections showing the localizations of GFP-ChEC36 **(A)** and GFP-ChEC39 **(B)** expressed transiently in *N. benthamiana* leaf cells. Both fusion proteins label punctate structures in the plant cytoplasm that resemble plant organelles, but those labelled by GFP-ChEC36 appear smaller on average than those labelled by GFP-ChEC39. Bars = 5 µm.
