# Supplementary material for: Subcellular Localization Screening of Colletotrichum higginsianum Effector Candidates Identifies Fungal Proteins Targeted to Plant Peroxisomes, Golgi Bodies, and Microtubules
Source: Front Plant Sci. 2018 May 2;9:562. doi: 10.3389/fpls.2018.00562 (PMC5942036; doi:10.3389/fpls.2018.00562)
Supplement: Supplementary file 5 [file Image_2.PDF]

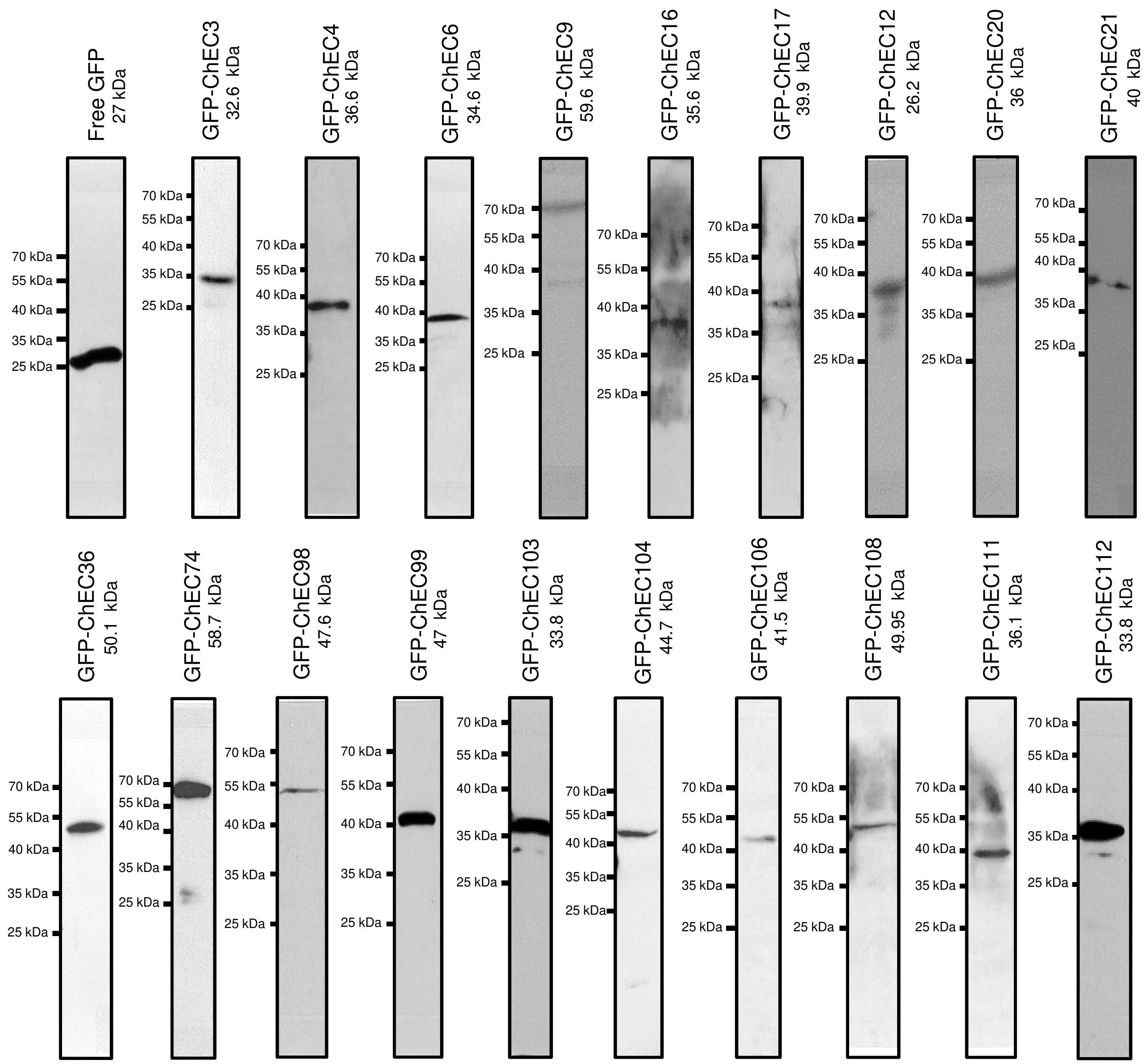

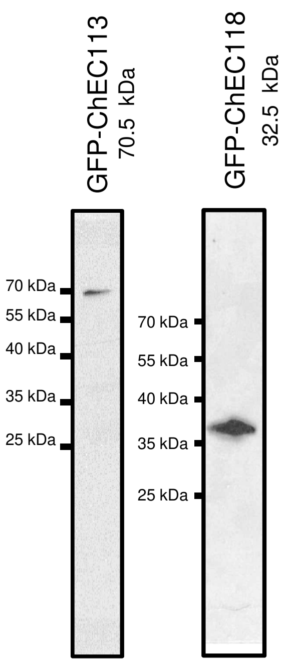

Supplementary Figure 2: Immunoblot analysis of GFP-tagged *C. higginsianum* effector candidates (ChECs) expressed transiently in *N. benthamiana*. Proteins were extracted from leaves two days after infiltration with Agrobacteria harboring the 35S::GFP-ChEC constructs. For each blot, the protein size ladder is indicated in kDa. The integrity of the GFP-fusion proteins was confirmed by probing the blots with anti-GFP antibodies.
